# Assessment of a Novel Adult Mass-Rearing Cage for *Aedes albopictus* (Skuse) and *Anopheles arabiensis* (Patton)

**DOI:** 10.3390/insects11110801

**Published:** 2020-11-13

**Authors:** Hamidou Maïga, Wadaka Mamai, Nanwintoum Séverin Bimbilé Somda, Thomas Wallner, Bèwadéyir Serge Poda, Gustavo Salvador-Herranz, Rafael Argiles-Herrero, Hanano Yamada, Jérémy Bouyer

**Affiliations:** 1 Insect Pest Control Laboratory, Joint FAO/IAEA Division of Nuclear Techniques in Food and Agriculture, International Atomic Energy Agency, P.O. Box 100 A-1400 Vienna, Austria; W.Mamai@iaea.org (W.M.); N.S.Bimbile-Somda@iaea.org (N.S.B.S.); T.Wallner@iaea.org (T.W.); R.Argiles-Herrero@iaea.org (R.A.-H.); H.Yamada@iaea.org (H.Y.); J.Bouyer@iaea.org (J.B.); 2 Institut de Recherche en Sciences de la Santé, Direction Régionale de l’Ouest, 01 BP 545 Bobo-Dioulasso, Burkina Faso; sergepoda71@yahoo.fr; 3 Institut de Recherche Agricole pour le Développement, P.O. Box 2123 Yaounde, Cameroun; 4 Technical School of Design, Architecture and Engineering, University CEU Cardenal Herrera, C/San Bartolomé 55, 46115 Alfara del Patriarca, Valencia, Spain; gsalva@uchceu.es

**Keywords:** sterile insect technique, insect cage, plexiglass, egg production, adult index, genetic control, dengue, malaria

## Abstract

**Simple Summary:**

*Aedes albopictus* (Asian tiger mosquito) and *Anopheles arabiensis* mosquito species can transmit several pathogens, including viruses and parasites which are the causative agents of diseases such as dengue, chikungunya, yellow fever, Zika and malaria, respectively. The use of insecticides against mosquito vectors has shown its limits. Proper management of these mosquito vectors is critical to prevent and/or control dengue outbreaks and malaria. Therefore, complementary vector control methods such as the sterile insect technique are envisioned. The potential to suppress mosquito populations by applying the sterile insect technique has been demonstrated in several countries. However, the technique, which relies on the mass-production of sterile males, needs innovations in mosquito mass-rearing techniques, including the development of low-cost adult cages. Here, we assessed the suitability of a new adult cage for mass egg production and egg quality for *Aedes albopictus* or *Anopheles arabiensis* mosquito species. Our results show the potential of the new cage for a mass production of high-quality eggs required for a sterile insect technique program targeting these mosquito species. Although the new cage has shown several advantages, further improvements are necessary to achieve economic efficiency and high production rates.

**Abstract:**

Successful implementation of the sterile insect technique (SIT) against *Aedes albopictus* and *Anopheles arabiensis* relies on a continuous supply of sterile males. To meet this requirement, optimization of the mass-rearing techniques is needed. This study, therefore, aims to assess a new mass-rearing cage (MRC) in terms of egg production efficiency and egg hatch rate (quality). In addition, adult survival was evaluated based on a cage adult-index for *Ae. albopictus*. Moreover, the cage’s suitability for use in mass *An. arabiensis* egg production was compared to that of the FAO/IAEA *Anopheles* reference cage. In *Ae. albopictus* rearing, the new MRC produced 1,112,110 eggs per cage following six blood meals, with minimum loss of eggs in the egging water. Furthermore, the adult index gave a good proxy of daily mortality rates in *Ae. albopictus*. In *An. arabiensis* rearing, about 130,000 eggs per egg batch were collected both from the new and the reference MRC. These findings suggest that the new MRC prototype is efficient in terms of egg production and can be used for mass-rearing in SIT programs targeting *Ae. albopictus* as well as *An. arabiensis*. The adult index was also positively validated for the detection of unusual mortality rates in *Ae. albopictus* mass-rearing facilities. Overall, the new MRC has shown several advantages; however, further improvements are necessary to minimize escapes during the egg collection processes.

## 1. Introduction

Both the Asian tiger mosquito *Aedes albopictus* (Skuse) and the yellow fever mosquito *Aedes aegypti* (Linnaeus) are highly invasive mosquito species of medical importance [1,2]. They can transmit several arboviral pathogens, which are the causative agents of diseases such as dengue, chikungunya, yellow fever, and Zika [3]. For instance, an estimated annual 390 million infections with dengue viruses including 96 million symptomatic cases were recorded in recent decades [4]. African malaria vectors including *Anopheles arabiensis* and the parasites they transmit also remain a serious threat to world health. Tremendous progress has been made over the last decade in reducing malaria-related morbidity and mortality to 405,000 worldwide deaths in 2018 [5], mainly attributable to significant upscaling of vector-control tools, including the use of long-lasting insecticidal nets (LLINs) and indoor residual spraying (IRS) [6].

The heavy reliance on insecticides to control adult populations of *Aedes* (especially during disease outbreaks) and *Anopheles* mosquitoes has led to the emergence of widespread resistance to the most commonly used compounds [7,8], making chemical-control-based strategies alone inadequate for the suppression of the numerous vector populations. Proper management of these mosquito vectors is critical to prevent and/or control dengue outbreaks and malaria. Therefore, complementary vector control methods such as the sterile insect technique (SIT) are needed in the frame of integrated vector management [9] to enhance existing efforts [10]. According to the International Standards for Phytosanitary Measures No. 5 Glossary of phytosanitary terms, the SIT is a “method of pest control using area-wide inundative releases of sterile insects to reduce reproduction in a field population of the same species”. Sterile insects have also been defined as beneficial organisms by the International Plant Protection Convention. The SIT has been applied successfully in more than 30 countries worldwide for various pest management strategies including suppression, eradication, containment, or prevention and is a species-specific and environment-friendly pest population control method [11].

The current production of sterile fruit flies supporting plant pest control programs amounts to over 3 billion insects per week [11,12]. In contrast, most mosquito-rearing facilities dedicated to SIT programs are currently occupied with developing improved tools and methods to enhance the capacity for mass-rearing the local mosquito strains in adequate numbers and are often limited by the inadequate size of readily available mosquito cages, which are commonly 30 × 30 × 30 cm or 60 × 60 × 60 cm [13,14] for small-scale releases. However, the production capacity of mass-rearing insectaries for mosquitoes has been increasing rapidly in recent years. Mass-rearing and release facilities for *Aedes* mosquitoes are currently being built in several countries, and technical and economic decision-making guidelines associated with facility design, cost, construction, equipment, and operation have been developed [15,16,17].

The potential to suppress *Ae. albopictus* mosquito populations by applying the SIT has been demonstrated in a feasibility study in Italy [18] and another in China, where the SIT was combined with the incompatible insect technique (IIT) [19]. A feasibility assessment of an area-wide integrated pest management (AW-IPM) program with an SIT component against *Ae. albopictus* is also ongoing on La Réunion island [20,21] and in Mauritius [22]. Moreover, the invasion of Europe by this species has triggered the development of a vector control management plan [23], and several countries, including Albania, Greece, Germany, Montenegro, and Spain, have initiated pilot trial releases of sterile males against *Ae. albopictus* recently [9]. A pilot project for SIT as a malaria vector control strategy against the outdoor biting *An. arabiensis* is also ongoing in South Africa [24]. Successful implementation of such projects relies on ensuring high levels of mosquito production and repeated releases of sterile males in overflooding numbers [25] that outcompete their wild counterparts within the target area [11,19]. To meet these requirements, novel methods and equipment including automatization of processes for the mass-rearing, sex separation, and release of mosquitoes are under development for deployment in the field [26]. Optimization of the mass-rearing conditions to produce eggs requires continuous efforts to achieve economic efficiency and high production rates. Presently, several mosquito-rearing methods, including a Food and Agricultural Organization/International Atomic Energy Agency (FAO/IAEA) stainless-steel reference mass-rearing cage (MRC) for *Anopheles* species (hereinafter referred to as reference *Anopheles* MRC), are available [27,28,29,30,31]. The capacity of the reference MRC to produce large numbers of eggs has been previously demonstrated [30,31]. However, the manufacturing cost of a single 2-m-long stainless-steel cage is high (approximately EUR 2300), and its weight of about 20 kg makes it cumbersome and costly to handle and ship. Zhang et al. [32] developed a cage structure for *Ae. albopictus* adults in support of the establishment of a medium-scale mosquito-rearing facility. However, egg collection was performed by opening the cages, which could ultimately increase the risk of escapes and thus bites by female mosquitoes. The Insect Pest Control Laboratory (IPCL) of the joint Food and Agricultural Organization/International Atomic Energy Agency (FAO/IAEA) Division of Nuclear Techniques in Food and Agriculture, Seibersdorf, Austria recently developed a low-cost MRC prototype made of plexiglass [33] to replace the previous expensive and heavy *Aedes* stainless-steel MRC [28]. The new MRC prototype, which can be fully operated from the outside without any direct contact with the adult mosquitoes, was successfully tested with *Ae. aegypti* and has been shown to perform well in terms of adult mosquito survival (based on adult index), egg production, and egg hatch rates, but it has not yet been tested for *Ae. albopictus* and *An. arabiensis*. This study, therefore, aimed to test and validate the cage in terms of egg production, egg hatch rate, and survival in *Ae. albopictus,* and to test the suitability of the cage for mass *An. arabiensis* egg production.

## 2. Materials and Methods

### 2.1. Mosquito Strains and Rearing Conditions

The *Ae. albopictus* strain used in this study originated from Italy (Rimini strain) and was transferred to the IPCL from the insectary of the Centro Agricoltura Ambiente, Bologna, Italy, in 2018. The immature stages were reared under controlled temperature, humidity, and light conditions (T = 28 ± 2 °C, 80 ± 10 RH%, and 14:10 h light/dark (L/D), including 1 h dawn and 1 h dusk) whereas adults were maintained in a separate room under 26 ± 2 °C, 60 ± 10 RH%, and 14:10 h light/dark, including 1 h dawn and 1 h dusk.

To test the cage, *Ae. albopictus* pupae were produced in the FAO/IAEA mass-rearing rack system [34]. A rearing density of 3.6 L1/cm^2^, corresponding to 18,000 first instar larvae (L1) in 5 L of reverse osmosis purified water per tray (5000 cm^2^ inner surface of the tray), was used following mass-rearing procedures developed at the IPCL [17,33,35]. A 4% FAO/IAEA black soldier fly based larval diet was added to each tray as follows: 100 mL on day 1, 200 mL on day 2, 400 mL (200 mL at 9 a.m. and 200 mL at 3 p.m.) on day 3, 0 mL on days 4 and 5 (weekends), and 200 mL on days 6 and 7, corresponding to 0.22, 0.44, 0.88, 0.0, 0.0, 0.22, and 0.22 mg of ingredients per larva per day, respectively.

The *An. arabiensis* (Dongola) strain was sourced from the Northern State of Sudan (Tropical Medicine Research Institute, Khartoum) and has been maintained at the IPCL since 2005. Larvae were reared using the mass-rearing rack system, in which the trays were filled with 4 L of deionized water and seeded with 4000 eggs per tray. Larvae were reared in a climate-controlled room maintained at 30 ± 1 °C and 70 ± 10% RH and fed with a 1% FAO/IAEA larval diet according to IAEA *Anopheles* mass-rearing protocols [36,37]. Adults were maintained under controlled temperature, RH, and light regimes (27 ± 1 °C, 70 ± 10% RH, and 12:12 (h) L/D light cycle with 1 h periods of simulated dawn and dusk).

### 2.2. Experimental Design to Assess the New Mass-Rearing Cage for Ae. albopictus

**Figure 1:**
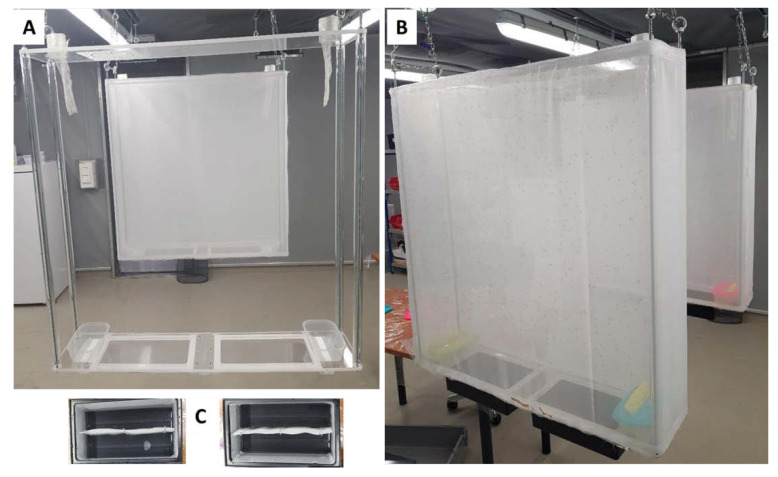
The structure of new mass-rearing cage (90 (L) × 90 (H) × 20 (W) cm) with the lower and the upper parts made of 1-cm-thick plates (**A**); the set-up with netting fixed with Velcro (VELCRO^®^ Freiberg am Neckar, Germany) (**B**); egg papers lining the walls of two black containers that can be removed laterally (**C**).

The new MRC prototype (90 (L) × 90 (H) × 20 (W) cm) [33] was modified in several ways to measure its efficiency for *Ae. albopictus* rearing while minimizing handling processes. The structure of the cage was stabilized by the four rods in the corners of the cage (Figure 1 and Supplementary Materials).

The upper and lower plates were made of a single 1-cm-thick PMMA plate instead of three layers as it had been previously. To ease removing and attaching the netting onto the cage frame, a hook-and-loop fastener (VELCRO^®^, Freiberg am Neckar, Germany)was added around the upper and the lower 1-cm-thick plexiglass cage components. In addition, the number of egg papers was increased to reduce the number of floating eggs in the water, and egg collection containers were now able to be removed from the side (laterally) instead of along the bottom of the cage (longitudinally).

The experimental design was similar to the one described by Maiga et al. [33]. Five cages (3 and 2 cages in parallel for the first and the second set of experiments, respectively) were each loaded with around 13,333 female and 4444 male *Ae. albopictus* pupae (female-to-male ratio of 3:1) over 2 consecutive days (Table 1), allowing the total final stocking of the cage with 12,000 and 4000 adult females and males respectively, before the first blood feeding (considering a 90% adult survival rate).

The schedule includes only days with tasks such as pupal loading, blood feeding, addition of egg papers, and egg paper removal (called egg collection). It starts with the first day of loading pupae and continues up to the fifth egg collection, covering a total active period of 27 days. Two egg collection events occurred in weeks 1 and 2, and one egg collection occurred in week 3.

All cages were hung from the ceiling of the adult rearing room. The pupae were loaded by sliding the containers from the side of the cage bottom and adding the pupae. Table 1 shows other tasks/events including 6 blood feedings (see [33] for more details), 5 egg paper insertion-events (each involving the addition of 4 egg papers, 1 on each of the 4 sides of the container and 1 in the middle, times two containers, amounting to 10 egg papers in total) (Figure 1C), and 5 egg paper removal events (including eggs laid directly in water (hereinafter “floating eggs”)) over the active period of 27 days. The number of eggs collected each week was estimated using an equation (Weight of eggs (mg) = (0.007 × Number of counted eggs) + 3.0143) [37]. The mean number of eggs per female was subsequently estimated based on the initial female pupae count.

The eggs collected on the papers and from the water (floating eggs) were dried and stored over 14 days before hatching. Egg hatch rates were then assessed by taking 3 samples of 100–150 eggs per cage and transferring them into 50 mL Falcon tubes filled with 40 mL of hatching solution (nutrient broth + yeast) [38] where they were left overnight to allow enough time for hatching. The following day, eggs were randomly taken from each Falcon tube and transferred to a petri dish; from a sample of 100 eggs, the numbers of hatched and nonhatched eggs were counted under a stereomicroscope. Egg hatch was assessed once per week using eggs from the first batch of eggs collected each week.

A constant daily mortality rate of the mosquitoes was assessed in the new MRC prototype by using the adult index (number of mosquitoes counted by square), a technique previously described by Maiga et al. [33] for *Ae. aegypti* as being an appropriate proxy for mortality assessment. Six 10 × 10 cm squares (3 on each cage side) were drawn onto the netted sides of MRCs using a fine marker. The numbers of male and female mosquitoes that were resting within each of the squares at a given point of time each day were counted for 4 weeks (24 days). Using the index only, adult *Ae. albopictus* daily mortality rates were estimated within a 24-day period for 3 MRCs.

### 2.3. Experimental Design to Assess the New Mass-Rearing Cage for Anopheles arabiensis

**Figure 2:**
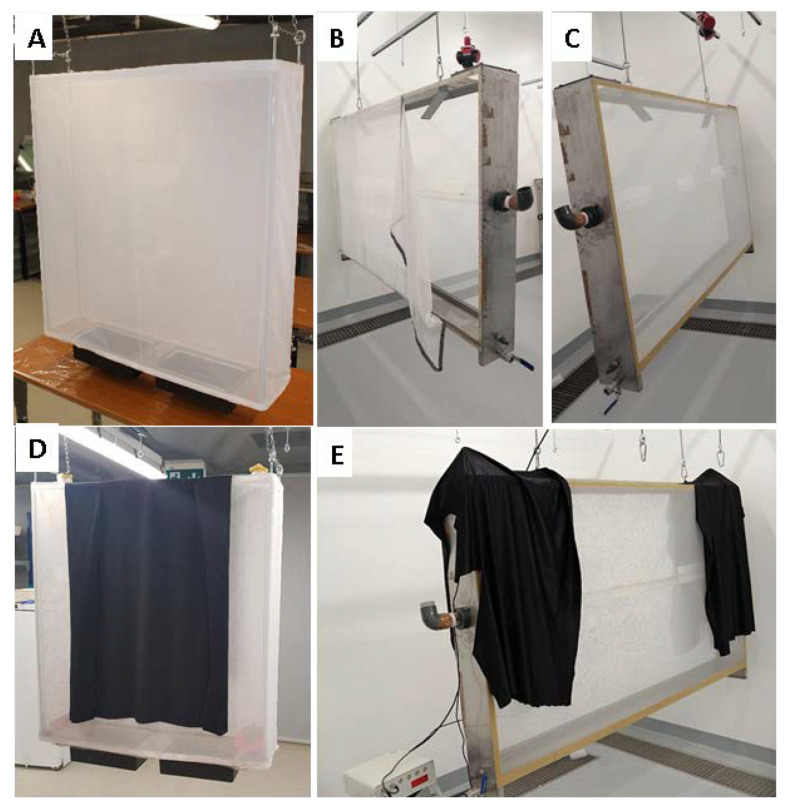
External view of the new mass-rearing cage prototype (90 (L) × 90 (H) × 20 (W) cm) (**A**) and the reference *Anopheles* mass-rearing cage (200 (L) × 100 (H) × 20 (W) cm) structure (**B**,**C**). The new mass-rearing cage (**D**) and the reference *Anopheles* mass-rearing cage (**E**) were covered with black cloth to enhance blood feeding.

Three new MRCs (Figure 2) were loaded with 15,000 mosquito pupae (estimated female-to-male ratio of 1:1) and were reared following the experimental design including pupal loading, blood feeding with defrosted bovine blood, sugar feeding, and egg collection (Table 2).

The schedule includes only days with tasks such as pupal loading, blood feeding, adding water to the cage for egg laying and egg collection, starting with the first day of loading pupae, up to the third egg collection (3 egg batches), covering a total active period of 17 days.

Female mosquitoes were blood-fed using a Hemotek membrane feeding system (Discovery Workshop, Lancashire, UK) with a modified (larger) heated plate placed on the top-center of the cage following the protocols developed at the IPCL [15]. The cages were hung from the ceiling of the adult rearing room and were three-fourths covered with a black cloth to create an artificial resting site that would stimulate blood feeding during the day [30].

The pupae quantification and loading procedures followed the protocol previously described for *Ae. aegypti* [33]. Three reference MRCs (200 (L) × 100 (H) × 20 (W) cm) for *Anopheles* were also loaded with a similar quantity of pupae derived by following the same rearing protocol. However, given the size of the reference *Anopheles* MRC, blood meals were offered using 2 Hemotek plates placed at each end of the cage (see [30] for more details). Eggs were collected 3 times (in 3 batches) over the 17 days of the cage rearing period. Eggs were air-dried in laboratory conditions, and the egg numbers per batch were estimated using the following equation: Weight of eggs (mg) = (0.00399 × Number of counted eggs) + 0.536, as described by Maiga et al. [36]. The mean number of eggs per female was subsequently estimated based on the initial female pupae count.

### 2.4. Data Analysis

All statistical analyses were performed in R (version 4.0.3) [39] using RStudio (RStudio, Inc. Boston, MA, USA, 2016). Generalized linear mixed models (glmer function in *lme4* package) were used with the appropriate distribution family and cage as a random factor for all analyses, considering inferences needed to be done independently of their levels in our specific experimental design [40].

The number of eggs per female was analyzed with Poisson errors. For *Ae. albopictus,* week of egg collection (3 levels: weeks 1–3) was considered as a fixed factor. For *An. arabiensis,* egg batch (3 levels: batches 1–3), cage type (2 levels: plexiglass and stainless-steel cages), and their interactions were considered as fixed factors.

The proportion of floating eggs and the egg hatch rate were analyzed using binomial errors. The proportion of floating eggs was analyzed as a function of the week of egg collection and the egg hatch rate was analyzed with week of egg collection, egg origin (2 levels: floating eggs and eggs collected on the papers), and their interactions considered as fixed factors.

The estimated constant mortality of adult mosquitoes was analyzed as a function of mosquito sex with a Gaussian distribution. To predict the daily mortality rates of adult *Ae. albopictus* from the counts in the squares on the new MRCs, a constant mortality rate was estimated from plotting the logarithm of counts against time [41]. A mathematical equation y = a + bx (y = ln (counts), x = time, a, b = coefficients) was generated, and the exponential of the coefficient “b” equaled the survival rate estimated from each square. The daily mortality from each square was then calculated as “1 – survival” (see data in the Supplementary Materials for more details).

For the validation, the full models were checked for overdispersion (using Bolker’s function [42]) and for normality and homogeneity of variances on the residuals [43]. Model simplification used the stepwise removal of terms, followed by likelihood ratio tests (LRTs). Term removals that significantly reduced explanatory power (*p* < 0.05) were retained in the minimal adequate model [44]. Differences between the levels of significant fixed factors were analyzed using post hoc Tukey tests (glht function in package *multcomp*) [45]. The significant interactions were analyzed using the emmeans function (in package *emmeans*) [46]. All means are provided with their standard error (S.E.), and all percentages are provided with their 95% confidence interval (95% C.I.).

## 3. Results

### 3.1. Egg Production, Floating Eggs, and Egg Hatch Rate for Aedes albopictus

**Figure 3:**
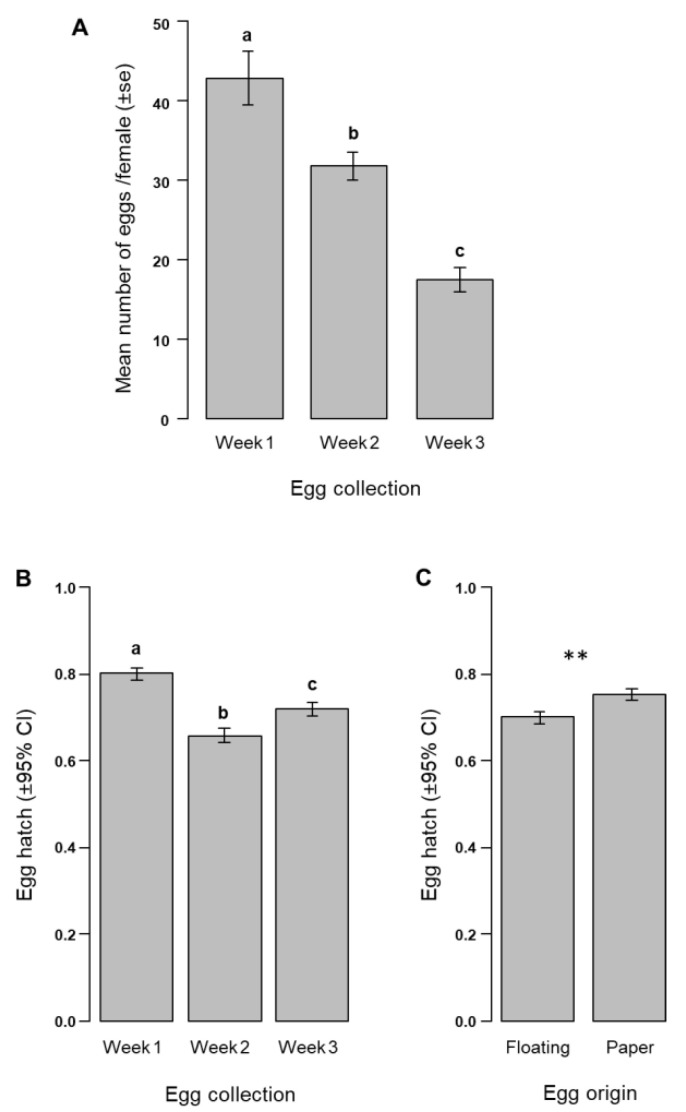
*Aedes albopictus* egg production and egg hatch. (**A**) Mean number of eggs per female according to the week of egg collection. Egg hatch according to the (**B**) week of egg collection and (**C**) egg origin. Different letters above the bars indicate significant differences (post hoc Tukey test, *p* < 0.05). ** indicates a significant difference (likelihood ratio test (LRT), *p* < 0.01).

A greater number (mean ± S.E.) of eggs per initial female were collected in the first week (43 ± 3) compared to the second (32 ± 1) and the third (17 ± 1) weeks (χ^2^ = 55.6, df = 2, *p* < 0.001; Figure 3A), corresponding to a mean number (±S.E.) of 1,112,110 ± 79,034 eggs collected per cage over the 3 weeks after a total of six blood meals. About 80% of the eggs were harvested during the first 2 weeks, and a total of 92 eggs were collected per female during the cage rearing duration.

Overall, the proportion of floating eggs collected from the *Ae. albopictus* MRCs was about 4.03 ± 0.01% (±95% C.I.) and was not significantly different between the weeks of collection (Weeks 1–3: 4.04 ± 0.02%, 3.94 ± 0.02%, and 4.18 ± 0.03%, respectively; χ^2^ = 0.28, df = 2, *p* = 0.86).

The egg hatch was significantly lower for the floating eggs compared to the eggs collected on papers (χ^2^ = 6.81, df = 1, *p* = 0.009; Figure 3C and Table 3). Overall, 72.59 ± 9.21% (±95% C.I.) of egg hatch was obtained regardless of the origin of the eggs (paper or floating) and was significantly greater in the first week of egg collection compared to the other weeks (χ^2^ = 36.52, df = 2, *p* < 0.001; Figure 3B and Table 3). Surprisingly, there was a significantly higher egg hatch in the third week than that of the second week (Figure 3B). The week × egg origin interaction had no effect on egg hatch (χ^2^ = 2.56, df = 2, *p* = 0.27).

### 3.2. Adult Aedes albopictus Mortality Rates

When the adult index was used to assess the mortality rates of *Ae. albopictus* in the new MRC prototypes, a mean constant daily mortality of 11.45 ± 1.30% (± S.E.) was observed. There was no sex bias in daily mortality of adult mosquitoes in the new MRCs (male: 11.36 ± 1.62%, female: 11.54 ± 0.92%, χ^2^ = 0.057, df = 1, *p* = 0.81).

### 3.3. Egg Production in the New Mass-Rearing Cage for Anopheles arabiensis

**Figure 4:**
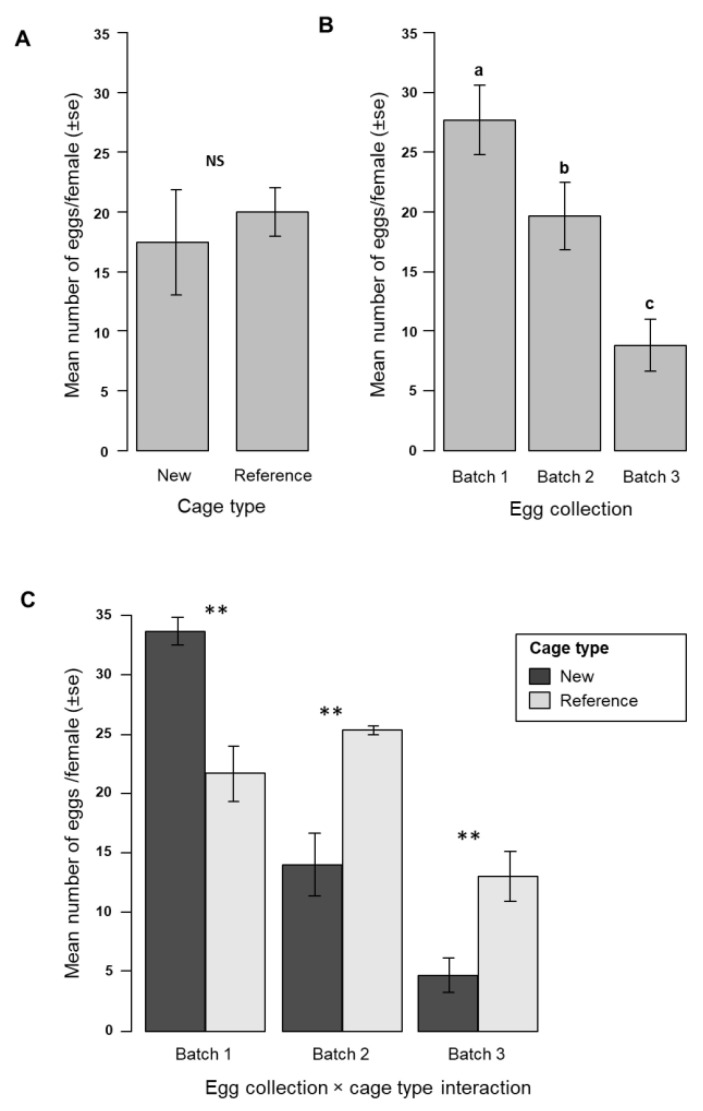
*Anopheles arabiensis* egg production. Mean number of eggs per female *Anopheles arabiensis* according to the (**A**) cage type (New, Reference), (**B**) collected egg batch, and (**C**) interaction between cage type and egg batch. Different letters above the bars indicate significant differences (post hoc Tukey test, df = 2, *p* < 0.05). ** indicates a significant difference (*p* < 0.01, interaction analyzed with Tukey test). NS stands for nonsignificant difference.

Overall, the total number of eggs collected in the new MRC made of plexiglass (mean ± S.E. = 394,417 ± 115,736) was similar compared to that of the reference Anopheles MRC (mean ± S.E. = 422,525 ± 114,833), corresponding to the mean numbers (±S.E.) of 52.6 ± 15.4 and 56.3 ± 15.3 eggs per female, respectively, after three egg-collections over the 17 days of the cage rearing duration. The mean number of eggs per female per egg batch was similar in the two types of MRC (New: 17.44 ± 4.37, Reference: 20 ± 2.04, χ^2^ = 1.09, df = 1, *p* = 0.29; Figure 4A). However, the mean number of eggs per female decreased significantly according to the egg collection batch (χ^2^ = 45.24, df = 2, *p* < 0.001; Figure 4B). There was a significant interaction between cage type and egg batch (χ^2^ = 26.91, df = 1, *p* < 0.001; Figure 4C). Moreover, the new MRC produced fewer eggs per female in the second and the third egg collections as compared to the reference *Anopheles* MRC (Figure 4C).

## 4. Discussion

The aim of this study was to investigate the overall efficiency of the new MRC prototype for mass-rearing *Ae. albopictus* as well as its suitability for *An. arabiensis* mass-rearing compared to the reference *Anopheles* stainless-steel cage. Our data show that the new MRC prototype is conducive to high egg yield of high quality with minimal presence of floating eggs. In addition, the adult index provided a good proxy of a daily mortality rate in *Ae. albopictus*. Similar numbers of *An. arabiensis* eggs were collected in the new MRC prototype as compared to the reference *Anopheles* MRC.

The egg yield for *Ae. albopictus* from the new MRC prototype confirmed previous results for the mass-rearing of its sister species, *Ae. aegypti* [33]. This shows that the cage prototype meets the requirements for *Aedes* species to complete their life cycle. It is known that natural conditions should be simulated where possible to accommodate the biological needs of insects when they are reared artificially (see [28] and references therein). In our previous work on *Ae. aegypti*, we recommended a reduction of pupal loading events to homogenize adult age and blood feeding rates and thereby increase overall blood volume intake and thus the total egg yield. In this study, pupae were loaded on two consecutive days, and the first blood meal was offered to females on day 7, corresponding to an adult age range of 5 to 6 days. This may be the prime reason for the greater number of eggs collected in the first week of egg collection. In the current study, about 80% of the egg yield during the cage rearing duration was collected within 2 weeks, confirming the recommendations of Zhang et al. [32], who suggested a 2-week-cycle for *Ae. albopictus* in rearing cages in a medium-scale rearing facility to produce 61 eggs/female. Here, we obtained approximately 37.5 eggs per female for the same period, whereas a prior trial in the stainless-steel mass-production cage showed a reduced number of 16 eggs per female [28], suggesting that various factors can impact egg production. Body size is a good proxy of mosquito fitness. Larval rearing quality guarantees good pupal production and pupal size/weight [47], which ultimately leads to larger male and female mosquitoes. Larger females will ingest larger blood meals (blood volume) and lay larger egg batches [48]. We suggest that the egg production obtained here could also be linked to the accumulated reserves from the highly nutritious FAO/IAEA black soldier fly based larval diet used for rearing the larvae [49]. In addition, male size was associated with a 46% increase in fecundity in *Ae. albopictus*, but not in *Ae. aegypti* [14,50]. Damiens et al. [14] suggested that a medium mass-rearing program for *Ae. albopictus* could produce 35,000 eggs (28 eggs/female) per week using 30 × 30 × 30 cm cages stocked with 1500 adults (female-to-male ratio of 3:1). If upscaling is the goal, about 13 cages would be needed to reach a weekly production of 450,000 eggs (37.5 eggs/female), which can be obtained with a single new MRC prototype. Up to 10 million eggs can be produced per week using 23 MRCs hung side-by-side in a 50 m^2^ adult mass-rearing room. Using the new adult mass-rearing cage would therefore reduce the workload associated with blood feeding and egg collection and thus increase cost–time efficiency.

*Aedes* species are container breeders; they deposit their eggs mainly on a moist substrate or, alternatively, directly on the water surface to increase the probability of egg and larval survival (reviewed in [51]). The new MRC prototype tested with *Ae. Aegypti* generated a fraction of floating eggs of up to 41%, mainly harvested along the sides of egg collection containers [33]. Contrarily, significantly fewer floating eggs were collected in the water for *Ae. albopictus*. This is likely due to the increased availability of substrate surface area provided by the increased number of egg papers that cover all four inner walls of the egg containers. The in-water oviposition behavior of *Ae. aegypti* and *Ae. albopictus* was previously linked to the presence of other substrates in the egg containers or the color of the egg container itself [51]. Moreover, skip oviposition behavior was observed more in *Ae. aegypti* females than in *Ae. albopictus* ([52] and references therein). The role of the number of egg substrates made available to *Ae. albopictus* has also been emphasized by Wasserberg et al. [53], who showed that at higher egg densities, mosquitoes laid more eggs on a virgin substrate.

In our study, floating eggs had poor egg hatch rates as compared to the eggs collected on papers, corroborating results from our previous work with *Ae. aegypti* [33]. Egg collection and maturation processes are important for the quality of *Aedes* eggs. It is possible that eggs laid in water are more difficult to handle since they have to be separated from mosquito debris by sieving prior to the drying process, which could damage the eggs. However, among several studies, Soares et al. [54] found that floating eggs had an extremely low 2% hatch rate, while Madeira et al. [55] and Rey and O’Connell [52] have reported higher rates of 47–53% and 73–80%, respectively. It is also possible that floating eggs would need more drying time before hatching because of the prolonged contact with water.

Other factors such as blood source and anticoagulants used could also affect egg hatch. Although we relied on a weekly collection of porcine blood from a nearby slaughterhouse with sufficient quality certification, we found a significant decrease in egg hatch over the weeks. It has been shown that the above-mentioned factors influenced *Culex quinquefasciatus* fecundity and fertility (egg hatch) rates [56]. However, the main reason for egg production and egg hatch to decrease with gonotrophic cycles is probably female age and a related depletion of nutritional reserves [57,58].

A careful design of mass-rearing cages is of utmost importance to guarantee mosquito survival and egg production [59]. Cage density is expected to decrease with age of the mosquitoes post-emergence due to natural mortality [33,41]. The new MRC prototype was stocked with a similar number of mosquitoes as in our previous work with *Ae. aegypti* [33] for which an adult-index-based survival rate estimation was first demonstrated. Here, the high egg production rate in *Ae. albopictus* highlights a good adult survival rate throughout the caged duration. Insect survival and fecundity are negatively impacted under crowded conditions [59], and female mosquitoes generally live longer than their male counterparts [60]. A prior study has shown that a difference could be observed among populations with respect to reproduction and survival of *Ae. albopictus* [61]. The adult-index measurement could therefore be used to spot any unusual increase in mortality within rearing cages in a factory setting. Each laboratory may set its own standards for its own strains for routine monitoring based on adult indexes, which may be a useful quality control indicator.

The similar egg production achieved in the new MRC prototype and the *Anopheles* stainless-steel cage [30] emphasizes the possibility to lower the costs of production for *An. arabiensis*. For example, considering the difference in cost, the MRC prototype manufactured locally can reduce initial investments for equipment by more than EUR 350,000 compared to the stainless-steel cage in a facility producing 10 million sterile male *Ae. aegypti* per week [33]. A prior rearing experiment in the reference *Anopheles* MRC loaded with a similar number of pupae yielded about 40 eggs per female (five egg batches) [31], whereas 52 eggs per female (three egg batches) were produced in the new MRC in this study. Although *Aedes* and *Anopheles* species are different in their biology and behavior, their ability to colonize small habitats enables rearing methods such as blood feeding and egg collection to be adapted, allowing the use of the same rearing cage model for both species. The new MRC prototype has produced a greater number of eggs during the first egg collection batch as compared with the reference *Anopheles* MRC. The reduced size of the new MRC prototype, which is about half the size of the reference *Anopheles* MRC, seemed to be more conducive for females to readily access the blood meals and thus to produce more eggs. In this regard, Zhang et al. [32] have also shown that cage structure can affect *Ae. albopictus* egg production. However, the variability in egg yield between egg collection batches showed that the new MRC prototype has a serious drawback that warrants improvement before it can be recommended for use in an *Anopheles* mass-rearing facility. While black egg containers are suitable for *Aedes* species oviposition [51], they presented an attractive resting site for both male and female *An. arabiensis*. This led to a heterogeneous cage distribution with very crowded resting sites which likely impacted female survivorship negatively [59] and increased escapes during egg collection, strongly impacting the overall egg yield after the first egg collection event.

## 5. Conclusions

This study demonstrated the potential of the new MRC prototype for the mass production of high-quality eggs required for an SIT program targeting *Ae. albopictus* or *An. arabiensis*. The adult-index-based survival monitoring could also be used to spot any abnormal mortality rates in *Ae. albopictus* mass-rearing facilities. Although the new MRC has shown several advantages, further improvements are necessary to minimize escapes during the egg collection processes.

## Figures and Tables

**Table 1:** Rearing procedures for *Aedes albopictus* experiments.

Task/Day	1	2	3	7	9	13	16	20	23	27
Pupal Loading	x	x								
Blood Feeding				x	x	x	x	x	x	
Egg Papers					x	x	x	x	x	
Egg Paper Removal						x	x	x	x	x
Week of Egg Collection						1		2		3

**Table 2:** Rearing procedures for *Anopheles arabiensis* experiments.

Task/Day	1	2	6	7	8	9	10	13	14	16	17
Pupal Loading	x	x									
Blood Feeding			x	x	x	x		x	x		
Water						x		x		x	
Egg Collection							1		2		3

**Table 3:** Effects of week of egg collection and egg origin on egg hatch for *Aedes albopictus*.

Fixed Factors	Predictors/Fixed Factors	Odds Ratio (±95% CI)	Estimate	Std. Error	Z-Value	*p*-Value
	(Intercept)	3.84 (2.17–6.78)	1.34	0.28	4.64	<0.001
Week of egg collection (3 levels: Weeks 1–3)	Week 2	0.51 (0.36–0.73)	−0.65	0.17	−3.65	<0.001
	Week 3	0.62 (0.43–0.89)	−0.46	0.18	−2.60	0.009
Egg origin (2 levels: Floating and Paper)	Paper	1.62 (1.12–2.34)	0.48	0.18	2.59	0.009

## Supplementary Materials

The following are available online at https://www.mdpi.com/2075-4450/11/11/801/s1: Figure S1: Overall view of the new mass-rearing cage. Figure S2: Main assembly of the new mass-rearing cage. Figure S3: Bottom tray assembly of the new mass-rearing cage. Figure S4: Top tray assembly of the new mass-rearing cage. Figure S5: Bottom tray parts of the new mass-rearing cage. Figure S6: Top tray parts of the new mass-rearing cage. Figure S7: Container for egg collection of the new mass-rearing cage. Supplementary data: raw data and statistical analyses.
