# Supplementary material for: Assessment of a Novel Adult Mass-Rearing Cage for Aedes albopictus (Skuse) and Anopheles arabiensis (Patton)
Source: Insects. 2020 Nov 13;11(11):801. doi: 10.3390/insects11110801 (PMC7697024; doi:10.3390/insects11110801)
Supplement: Supplementary file 1 [file insects-11-00801-s001.zip › Supplementary Materials/Figure S3. Bottom_Tray_Assembly.pdf]

1

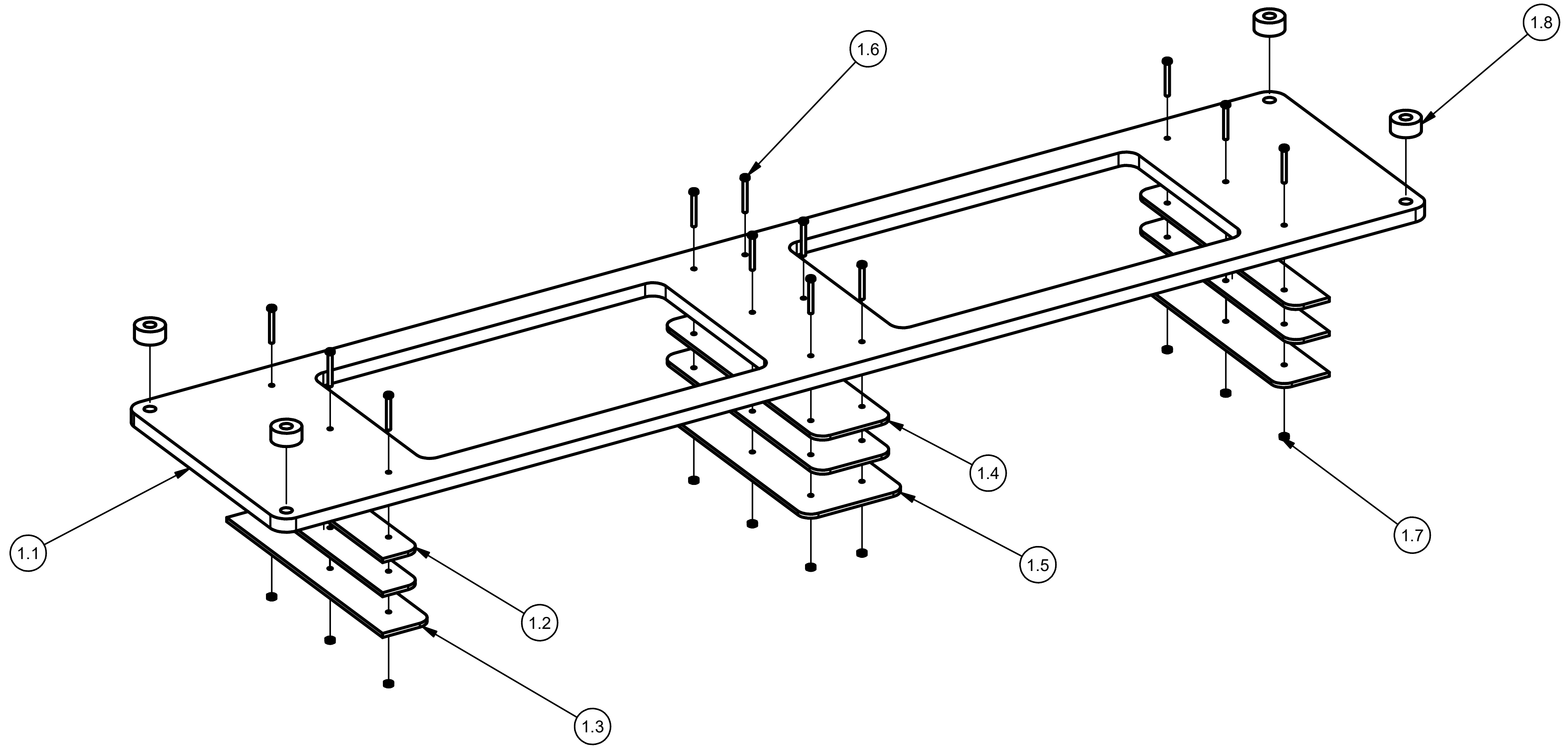

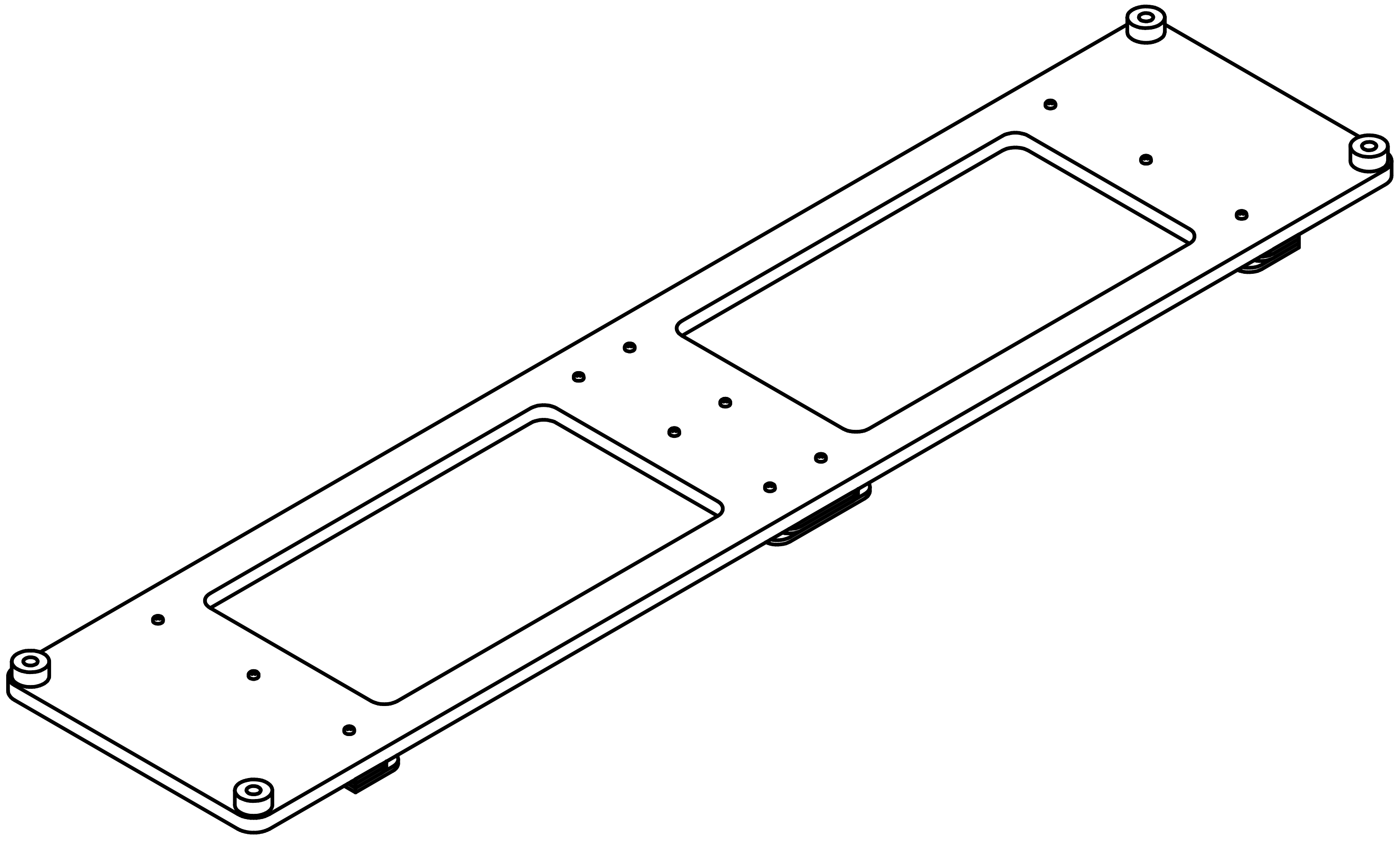

Item	Quantity	Part	Desxription
1.8	4	Tube center washer	10mm transparent PMMA
1.7	12	ISO 4032 - M3	Hex nuts
1.6	12	ISO 7045 - M3 x 25	Screws
1.5	1	Support center Part - big	3mm transparent PMMA
1.4	2	Support center Part - small	3mm transparent PMMA
1.3	2	Support Side Part - big	3mm transparent PMMA
1.2	4	Support side part - small	3mm transparent PMMA
1.1	1	Main board	10mm transparent PMMA

	Name	Date
Designed	G. Salvador-Herranz	2020/06/22
Revised	R. Argilés	2020/06/22

Joint FAO/IAEA Programme

Insect Pest Control Section

Scale: mm

Aedes Mass Rearing Cage

Bottom Tray Assembly

Number: AMRC_V1

Sheet: 3/7
