# Supplementary material for: Assessment of a Novel Adult Mass-Rearing Cage for Aedes albopictus (Skuse) and Anopheles arabiensis (Patton)
Source: Insects. 2020 Nov 13;11(11):801. doi: 10.3390/insects11110801 (PMC7697024; doi:10.3390/insects11110801)
Supplement: Supplementary file 1 [file insects-11-00801-s001.zip › Supplementary Materials/Figure S4. Top_Tray_Assembly.pdf]

2

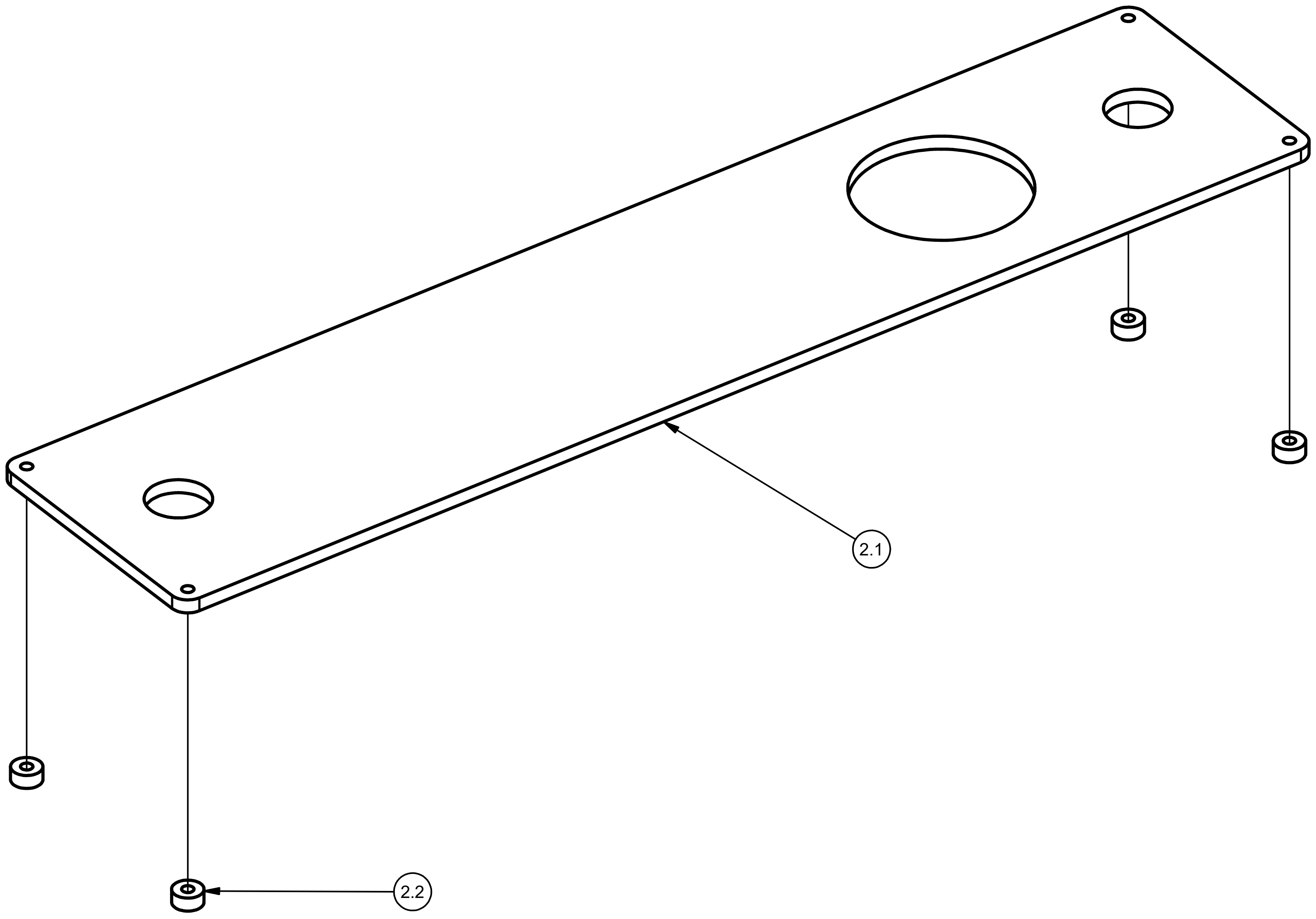

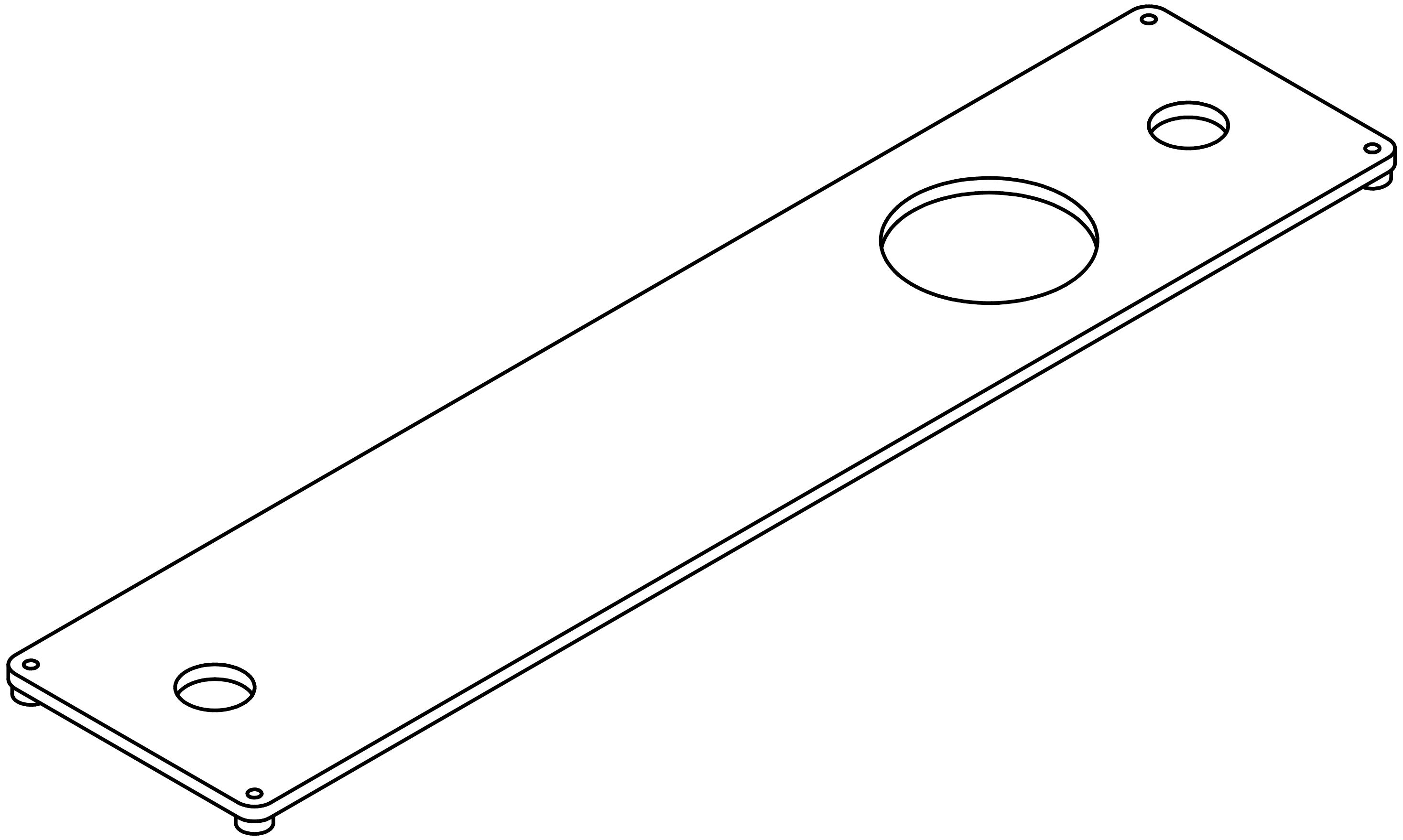

Item	Quantity	Part	Description
2.2	4	Tube center washer	10mm transparent PMMA
2.1	1	Main board	10mm transparent PMMA

	Name	Date
Designed	G. Salvador-Herranz	2020/06/22
Revised	R. Argilés	2020/06/22

Insect Pest Control Section

Scale: mm

Aedes Mass Rearing Cage

Top Tray Assembly

Number: AMRC_V1

Sheet: 4/7
