# Supplementary material for: Assessment of a Novel Adult Mass-Rearing Cage for Aedes albopictus (Skuse) and Anopheles arabiensis (Patton)
Source: Insects. 2020 Nov 13;11(11):801. doi: 10.3390/insects11110801 (PMC7697024; doi:10.3390/insects11110801)
Supplement: Supplementary file 1 [file insects-11-00801-s001.zip › Supplementary Materials/Figure S5. Bottom_Tray_Parts.pdf]

1.1

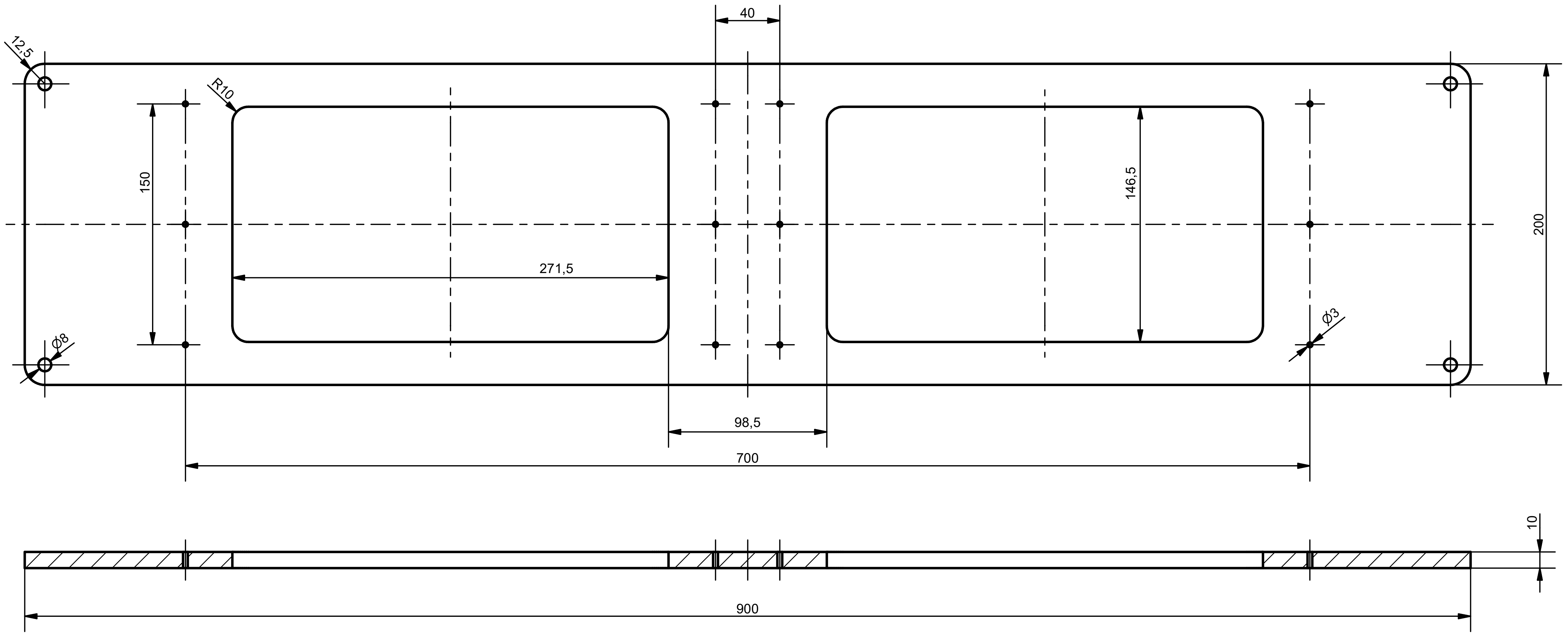

1.2

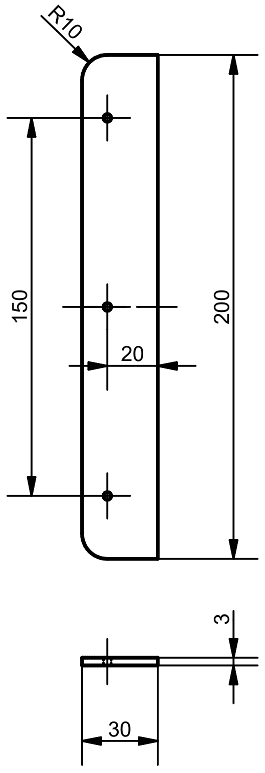

1.3

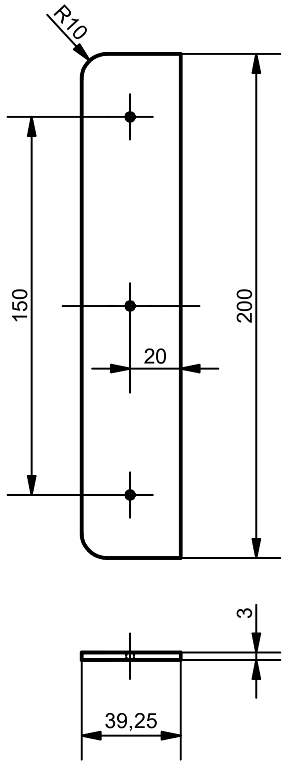

1.4

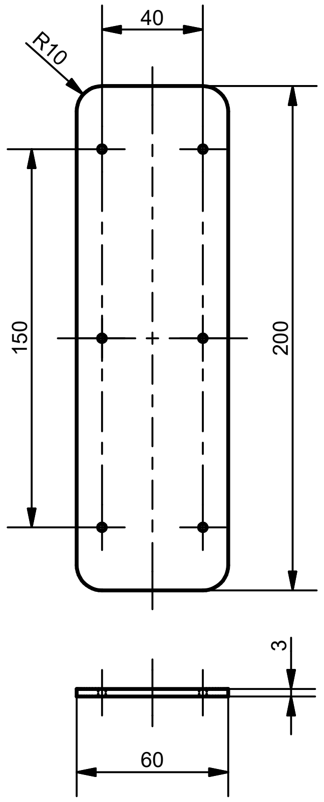

1.5

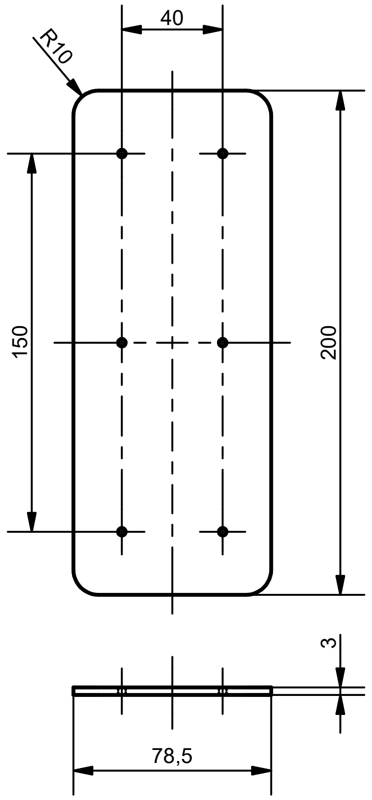

1.8

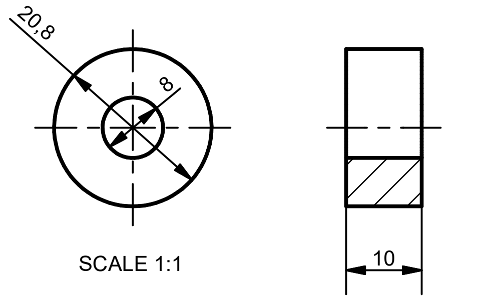

SCALE 1:1

	Name	Date
Designed	G. Salvador-Herranz	2020/06/22
Revised	R. Argilés	2020/06/22

Joint FAO/IAEA Programme

Insect Pest Control Section

Scale	Title	Number / Sheet
1:3 mm	Aedes Mass Rearing Cage<br>Bottom Tray - Parts	Number: AMRC_V1<br>Sheet: 5/7
