# Supplementary material for: Assessment of a Novel Adult Mass-Rearing Cage for Aedes albopictus (Skuse) and Anopheles arabiensis (Patton)
Source: Insects. 2020 Nov 13;11(11):801. doi: 10.3390/insects11110801 (PMC7697024; doi:10.3390/insects11110801)
Supplement: Supplementary file 1 [file insects-11-00801-s001.zip › Supplementary Materials/Figure S7. Container.pdf]

6

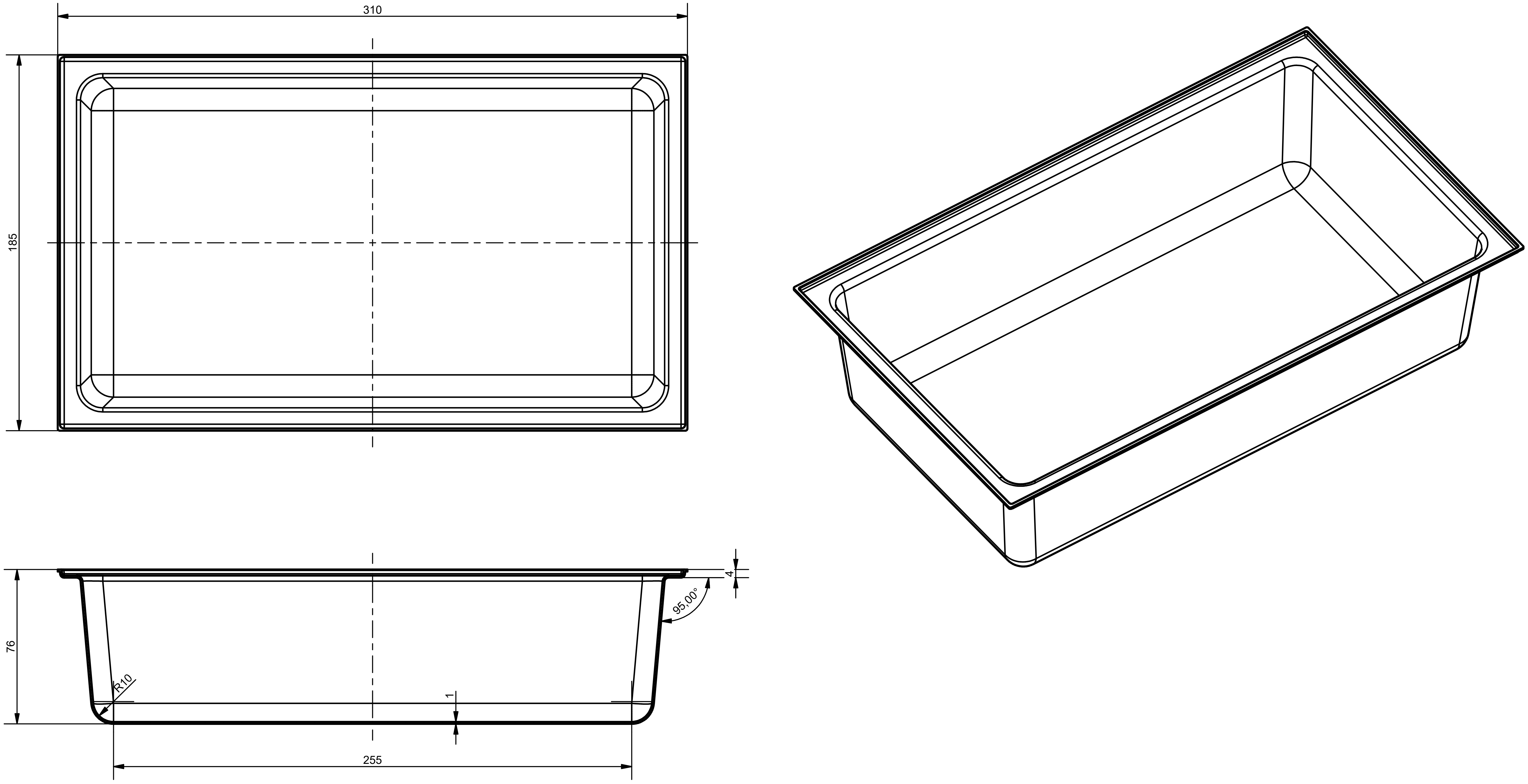

	Name	Date		
Designed	G. Salvador-Herranz	2020/06/22		Joint FAO/IAEA Programme
Revised	R. Argilés	2020/06/22		**Insect Pest Control Section**
Scale 1:2 mm	Aedes Mass Rearing Cage			Number AMRC_V1
	Container			Sheet 8/7
