# Supplementary material for: Assessment of a Novel Adult Mass-Rearing Cage for Aedes albopictus (Skuse) and Anopheles arabiensis (Patton)
Source: Insects. 2020 Nov 13;11(11):801. doi: 10.3390/insects11110801 (PMC7697024; doi:10.3390/insects11110801)
Supplement: Supplementary file 1 [file insects-11-00801-s001.zip › Supplementary Materials/Supplementary data_raw data and stats/Supplementary file_data analyses.pdf]

Supplementary file: SCRIPTS/OUTPUT: statistical analyses

Assessment of a novel adult mass-rearing cage for *Aedes albopictus* (Skuse) and *Anopheles arabiensis* (Patton)

Hamidou Maïga1,2*, Wadaka Mamai1,3, Nanwintoum Séverin Bimbilé Somda1,2, Thomas Wallner1, Serge Poda2, Gustavo Salvador Herranz4, Rafael Argiles Herrero1, Hanano Yamada1, Jeremy Bouyer1


```
###########################################################################
#############
###########################  1. Aedes albopictus egg production  #
###########################################################################
#############
#------------ Loading the dataset ---------------------#

data<-read.csv2("aedes.egg.production.csv", dec=".", header=TRUE)
str(data)

data <- transform(data,
                  replicate=factor(replicate),
                  cage=factor(cage),
                  cage2=factor(cage2),
                  week=factor(week))
str(data)
summary (data)
attach(data)

#------------ Statistical analysis ---------------------#

library(lme4)
mod <- lmer(npf2 ~ week + (1|cage2))
summary(mod)

par(mfrow=c(2,2))
hist(resid(mod),freq=FALSE, ylim = c(0,0.5))
lines(density(resid(mod)), col="red")
library(car)
qqPlot(resid(mod))
plot(fitted(mod),resid(mod))
abline(h=0)
shapiro.test(resid(mod))

mod <- glmer(npf2 ~ week + (1|cage2), family=poisson)
summary(mod)

overdisp_fun <- function(model) {
  rdf <- df.residual(model)
  rp <- residuals(model,type="pearson")
  Pearson.chisq <- sum(rp^2)
  prat <- Pearson.chisq/rdf
```

```
  pval <- pchisq(Pearson.chisq, df=rdf, lower.tail=FALSE)
  c(chisq=Pearson.chisq, rdf=rdf, ratio=prat ,p.val=pval)
}
overdisp_fun(mod)

mod2 <- update(mod,~.-week)
anova(mod,mod2)
library(multcomp)
summary(glht(mod,linfct = mcp(week="Tukey")))


#------------ Plots ----------------#

mean(npf2)
sd(npf2)/sqrt(nrow(data))

par(mfrow=c(1,2))
boxplot(npf2~week,ylab="Number of eggs produced/female", xlab="Egg
collection")
(m<-tapply(npf2,week,mean))
(se<-tapply(npf2,week,sd)/sqrt(table(week)))
bar<-barplot(m,beside=T,las=1,cex.axis=0.8,ylab="Mean number of eggs
produced/female (±se)", xlab="Egg collection",ylim=c(0,50))
arrows(bar,m+se,bar,m-se,angle=90,code=3,length=0.05)

######################################  END
####################################

############################################################################
#################
########################### 2. Aedes albopictus egg hatching rate
#########################
############################################################################
#################


#------------ Loading the dataset ---------------------#

data<-read.csv2("aedes.egg.hatching.csv", dec=".", header=TRUE)
str(data)
data <- transform(data,
                  replicate=factor(replicate),
                  cage=factor(cage),
                  cage2=factor(cage2),
                  week=factor(week),
                  eggorigin=factor(eggorigin),
                  eggsample=factor(eggsample))
str(data)
summary (data)
attach(data)


#------------ Statistical analysis ---------------------#


y <- cbind(hatched,unhatched)

library(lme4)
mod <- glmer(y ~  week*eggorigin + (1|cage2), family = "binomial")
summary(mod)
```

```
overdisp_fun <- function(model) {
  rdf <- df.residual(model)
  rp <- residuals(model,type="pearson")
  Pearson.chisq <- sum(rp^2)
  prat <- Pearson.chisq/rdf
  pval <- pchisq(Pearson.chisq, df=rdf, lower.tail=FALSE)
  c(chisq=Pearson.chisq, rdf=rdf, ratio=prat ,p.val=pval)
}
overdisp_fun(mod)

data$obs<- 1:nrow(data)
str(data)
mod <- glmer(y ~  week*eggorigin + (1|cage2) + (1|obs), data=data, family =
"binomial")
summary(mod)
mod2 <- update(mod,~.-week:eggorigin)
anova(mod,mod2)
summary(mod2)
mod3 <- update(mod2,~.-eggorigin)
anova(mod2,mod3)
mod4 <- update(mod2,~.-week)
anova(mod2,mod4)
library(multcomp)
summary(glht(mod2,linfct = mcp(week="Tukey")))
summary(emmeans(mod2,pairwise~week), infer=TRUE, type="response")
summary(emmeans(mod2,pairwise~eggorigin), infer=TRUE, type="response")

#Full model
mod <- glmer(y ~  week*eggorigin + (1|cage2) + (1|obs), data=data, family =
"binomial")
summary(mod)

# OR
exp(cbind(OR=fixef(mod)))

#95% CI
exp(confint(mod,metho="Wald"))
#------------ Plots ----------------#

(prop<-sum(hatched)/sum(hatched+unhatched))
(IC<-1.96*sqrt(prop*(1-prop)/nrow(data)))

par(mfrow=c(1,2))
(p<-tapply(hatched,week,sum)/tapply(hatched+unhatched,week,sum))
(IC<-1.96*sqrt(p*(1-p)/tapply(hatched+unhatched,week,sum)))
bar<-barplot(p,beside=T,las=1,cex.axis=0.8,ylab="Egg hatch rate (±95%CI)",
             xlab="Egg collection", ylim=c(0,1), cex.names=0.9,cex.lab=1)
arrows(bar,p+IC,bar,p-IC,angle=90,code=3,length=0.05)

(p<-tapply(hatched,eggorigin,sum)/tapply(hatched+unhatched,eggorigin,sum))
(IC<-1.96*sqrt(p*(1-p)/tapply(hatched+unhatched,eggorigin,sum)))
bar<-barplot(p,beside=T,las=1,cex.axis=0.8,ylab="Egg hatch rate (±95%CI)",
             xlab="Egg origin", ylim=c(0,1), cex.names=0.9,cex.lab=1)
arrows(bar,p+IC,bar,p-IC,angle=90,code=3,length=0.05)

####################################  END
####################################

########################################################################
################
```

```
###########################  3. Aedes albopictus egg floating rate
#########################
############################################################################
#################


#------------dataset ---------------------#

data<-read.csv2("aedes.egg.floating.csv", dec=".", header=TRUE)
str(data)
data <- transform(data,
                  replicate=factor(replicate),
                  cage=factor(cage),
                  cage2=factor(cage2),
                  week=factor(week))
str(data)
summary (data)
attach(data)


#------------ Statistical analysis ---------------------#

y <- cbind(floating,paper)

library(lme4)
mod <- glmer(y ~  week + (1|cage2),family=binomial)
summary(mod)

overdisp_fun <- function(model) {
  rdf <- df.residual(model)
  rp <- residuals(model,type="pearson")
  Pearson.chisq <- sum(rp^2)
  prat <- Pearson.chisq/rdf
  pval <- pchisq(Pearson.chisq, df=rdf, lower.tail=FALSE)
  c(chisq=Pearson.chisq, rdf=rdf, ratio=prat ,p.val=pval)
}
overdisp_fun(mod)
data$obs<- 1:nrow(data)
str(data)
mod <- glmer(y ~  week + (1|cage2) + (1|obs), data=data, family =
"binomial")
summary(mod)
mod2 <- update(mod,~.-week)
anova(mod,mod2)


#------------ Plots ----------------#

(prop<-sum(floating)/sum(floating+paper))
(IC<-1.96*sqrt(prop*(1-prop)/sum(floating+paper)))

(p<-tapply(floating,week,sum)/tapply(floating+paper,week,sum))
(IC<-1.96*sqrt(p*(1-p)/tapply(floating+paper,week,sum)))
bar<-barplot(p,beside=T,las=1,cex.axis=0.8,ylab="Egg floating rate
(±95%CI)",
             xlab="Egg collection", ylim=c(0,1), cex.names=0.9,cex.lab=1)
arrows(bar,p+IC,bar,p-IC,angle=90,code=3,length=0.05)
```

```
######################################  END
####################################

###########################################################################
################
########################### 4.  Aedes adult mortality rate
#################################
###########################################################################
#################


#------------ Loading the dataset ---------------------#

data<-read.csv2("aedes.daily.mortality.csv", dec=".", header=TRUE)
str(data)
data$cage <- as.factor(data$cage)
data$square <- as.factor(data$square)
data$sex <- as.factor(data$sex)

str(data)
summary (data)
attach(data)

#------------ Statistical analysis ----------------------#

library(lme4)
mod <- lmer(dailymortality ~ sex + (1|cage))
summary(mod)
par(mfrow=c(2,2))
hist(resid(mod),freq=FALSE, ylim = c())
lines(density(resid(mod)), col="red")
library(car)
qqPlot(resid(mod))  # good for normality
plot(fitted(mod),resid(mod))
abline(h=0)  # good for variance
shapiro.test(resid(mod))    # p-value = 0.5383  good for normality
mod2 <- update(mod,~.-sex)
anova(mod,mod2)
#------------ Plots ----------------#

mean(dailymortality)
sd(dailymortality)/sqrt(3)
par(mfrow=c(1,2))
boxplot(dailymortality~sex,ylab="Constant daily mortality rate",
xlab="Sex")
(m<-tapply(dailymortality,sex,mean))
(se<-tapply(dailymortality,sex,sd)/sqrt((3)))
bar<-barplot(m,beside=T,las=1,cex.axis=0.8,ylab="Mean constant daily
mortality rate (±se)", xlab="Sex",ylim=c(0,0.16))
arrows(bar,m+se,bar,m-se,angle=90,code=3,length=0.05)

######################################  END
####################################################################???
```

```
################################################################################
#################
########  5. Anopheles arabiensis egg production
######################
################################################################################
#################


#------------ Loading the dataset ---------------------#

data<-read.csv2("anopheles.egg.production.csv", dec=".", header=TRUE)
str(data)
data <- transform(data,
                  cage=factor(cage),
                  week=factor(week),
                  eggbatch=factor(eggbatch),
                  cagetype=factor(cagetype))
str(data)
summary (data)
attach(data)

library(lme4)
mod <- lmer(npf2 ~ cagetype*eggbatch + (1|cage))
summary(mod)

par(mfrow=c(2,2))
hist(resid(mod),freq=FALSE, ylim = c(0,0.3))
lines(density(resid(mod)), col="red")
library(car)
qqPlot(resid(mod))
plot(fitted(mod),resid(mod))
abline(h=0)    # p-value = 0.3534  # good

mod <- glmer(npf2 ~ cagetype*eggbatch + (1|cage), family=poisson)
summary(mod)

overdisp_fun <- function(model) {
  rdf <- df.residual(model)
  rp <- residuals(model,type="pearson")
  Pearson.chisq <- sum(rp^2)
  prat <- Pearson.chisq/rdf
  pval <- pchisq(Pearson.chisq, df=rdf, lower.tail=FALSE)
  c(chisq=Pearson.chisq, rdf=rdf, ratio=prat ,p.val=pval)
}
overdisp_fun(mod)

#------------ Statistical analysis ---------------------#

mod2 <- update(mod,~.-cagetype:eggbatch)
anova(mod,mod2)
library(car)
Anova(mod,Type=III)

library(multcomp)
summary(glht(mod,linfct = mcp(eggbatch="Tukey")))
library(emmeans)
summary(emmeans(mod,pairwise~cagetype|eggbatch),infer=TRUE,
type="response")
```

```
#------------ Plots ----------------#


mean(neggs)
sd(neggs)/sqrt(nrow(data))

par(mfrow=c(1,2))
boxplot(npf2 ~ cagetype,ylab="Number of eggs produced/female", xlab="Cage
type")

(m<-tapply(npf2,cagetype,mean))
(se<-tapply(npf2,cagetype,sd)/sqrt(table(cagetype)))
bar<-barplot(m,beside=T,las=1,cex.axis=0.8,ylab="Mean number of eggs
produced/female (±se)", xlab="Cage type",ylim=c(0,35))
arrows(bar,m+se,bar,m-se,angle=90,code=3,length=0.05)

boxplot(npf2 ~ eggbatch,ylab="Number of eggs produced/female", xlab="Egg
batch")
(m<-tapply(npf2,eggbatch,mean))
(se<-tapply(npf2,eggbatch,sd)/sqrt(table(eggbatch)))
bar<-barplot(m,beside=T,las=1,cex.axis=0.8,ylab="Mean number of eggs
produced/female (±se)", xlab="Egg collection",ylim=c(0,35))
arrows(bar,m+se,bar,m-se,angle=90,code=3,length=0.05)

boxplot(npf2~cagetype:eggbatch, sep = "_", lex.order = TRUE, col =
c("gray25", "gray70","gray90"),ylab="Number of eggs produced by female",
xlab="Egg batch")
(m<-tapply(npf2,list(eggbatch,cagetype),mean))
(se<-
tapply(npf2,list(eggbatch,cagetype),sd)/sqrt(table(eggbatch,cagetype)))
bar<-barplot(m,beside=T,las=1,cex.axis=0.8,ylab="Mean number of eggs
produced/female (±se)", xlab=" Egg collection x cage type
interaction",ylim=c(0,35))
arrows(bar,m+se,bar,m-se,angle=90,code=3,length=0.05)
legend(6.6,38,levels(eggbatch), fill=c("gray25", "gray70","gray90"),xpd =
T)

(m<-tapply(npf2,list(cagetype,eggbatch),mean))
(se<-
tapply(npf2,list(cagetype,eggbatch),sd)/sqrt(table(cagetype,eggbatch)))
bar<-barplot(m,beside=T,las=1,cex.axis=0.8,ylab="Mean number of eggs
produced by female (±se)", xlab=" Egg collection : cage type
interaction",ylim=c(0,35))
arrows(bar,m+se,bar,m-se,angle=90,code=3,length=0.05)
legend(6.6,38,levels(cagetype), fill=c("gray25", "gray70","gray90"),xpd =
T)


#######################################  END
#############################################################################
######################
```

OUTPUT

```
> ############################################################################
###############
> ########################### 1.  Aedes albopictus egg production   ########
#################
> ############################################################################
###############
>
>
> #------------ Loading the dataset ---------------------#
>
> library(readr)
> data <- read_delim("statsmaiga/MRC optimization/Insect ms DATA/data_resub
m/aedes.egg.production.csv",
+                                     ";", escape_double = FALSE, trim_ws =
TRUE)
Parsed with column specification:
cols(
  replicate = col_character(),
  cage = col_character(),
  cage2 = col_character(),
  week = col_character(),
  npf = col_double(),
  npf2 = col_double()
)
> View(data)
>
> str(data)
tibble [15 x 6] (S3: spec_tbl_df/tbl_df/tbl/data.frame)
 $ replicate: chr [1:15] "R1" "R1" "R1" "R1" ...
 $ cage     : chr [1:15] "C1" "C2" "C3" "C1" ...
 $ cage2    : chr [1:15] "C1" "C2" "C3" "C1" ...
 $ week     : chr [1:15] "Week 1" "Week 1" "Week 1" "Week 2" ...
 $ npf      : num [1:15] 49.7 45.5 48.3 31 33 ...
 $ npf2     : num [1:15] 50 45 48 31 33 38 19 18 22 31 ...
 - attr(*, "spec")=
  .. cols(
  ..   replicate = col_character(),
  ..   cage = col_character(),
  ..   cage2 = col_character(),
  ..   week = col_character(),
  ..   npf = col_double(),
  ..   npf2 = col_double()
  .. )
> data <- transform(data,
+                   replicate=factor(replicate),
+                   cage=factor(cage),
+                   cage2=factor(cage2),
+                   week=factor(week))
>
> str(data)
'data.frame':  15 obs. of  6 variables:
 $ replicate: Factor w/ 2 levels "R1","R2": 1 1 1 1 1 1 1 1 1 2 ...
 $ cage     : Factor w/ 3 levels "C1","C2","C3": 1 2 3 1 2 3 1 2 3 1 ...
 $ cage2    : Factor w/ 5 levels "C1","C2","C3",..: 1 2 3 1 2 3 1 2 3 4 ...
 $ week     : Factor w/ 3 levels "Week 1","Week 2",..: 1 1 1 2 2 2 3 3 3 1
...
 $ npf      : num  49.7 45.5 48.3 31 33 ...
 $ npf2     : num  50 45 48 31 33 38 19 18 22 31 ...
> summary (data)
```

```
 replicate cage   cage2      week         npf              npf2       
 R1:9      C1:6   C1:3   Week 1:5   Min.   :14.20   Min.   :14.00  
 R2:6      C2:6   C2:3   Week 2:5   1st Qu.:20.69   1st Qu.:20.50  
           C3:3   C3:3   Week 3:5   Median :31.01   Median :31.00  
                  C4:3              Mean   :30.89   Mean   :30.67  
                  C5:3              3rd Qu.:39.41   3rd Qu.:39.00  
                                    Max.   :49.66   Max.   :50.00  
> attach(data)
> 
> #------------ Statistical analysis ----------------------#
> 
> library(lme4)
Le chargement a nécessité le package : Matrix
> mod <- lmer(npf2 ~ week + (1|cage2), data=data)
> summary(mod)
Linear mixed model fit by REML ['lmerMod']
Formula: npf2 ~ week + (1 | cage2)
   Data: data

REML criterion at convergence: 74.1

Scaled residuals: 
     Min       1Q   Median       3Q      Max 
-2.02481 -0.18359  0.02384  0.40967  1.55186 

Random effects:
 Groups   Name        Variance Std.Dev.
 cage2    (Intercept) 18.22    4.268   
 Residual             10.18    3.191   
Number of obs: 15, groups:  cage2, 5

Fixed effects:
            Estimate Std. Error t value
(Intercept)   42.800      2.383   17.96
weekWeek 2   -11.000      2.018   -5.45
weekWeek 3   -25.400      2.018  -12.59

Correlation of Fixed Effects:
           (Intr) wekWk2
weekWeek 2 -0.423       
weekWeek 3 -0.423  0.500
> 
> par(mfrow=c(2,2))
> hist(resid(mod),freq=FALSE, ylim = c(0,0.5))
> lines(density(resid(mod)), col="red")
> 
> library(car)
Le chargement a nécessité le package : carData
Registered S3 methods overwritten by 'car':
  method                          from
  influence.merMod                lme4
  cooks.distance.influence.merMod lme4
  dfbeta.influence.merMod         lme4
  dfbetas.influence.merMod        lme4
> 
> qqPlot(resid(mod))
[1] 10  1
> plot(fitted(mod),resid(mod))
> abline(h=0)
```

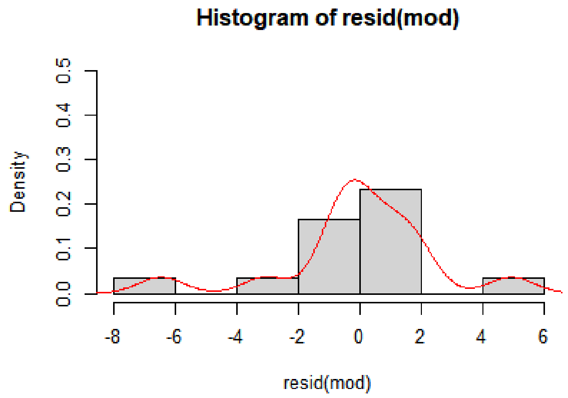

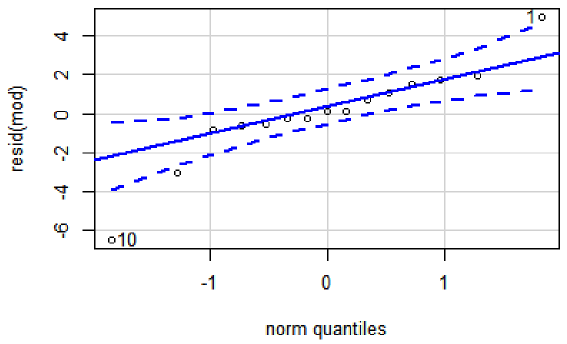

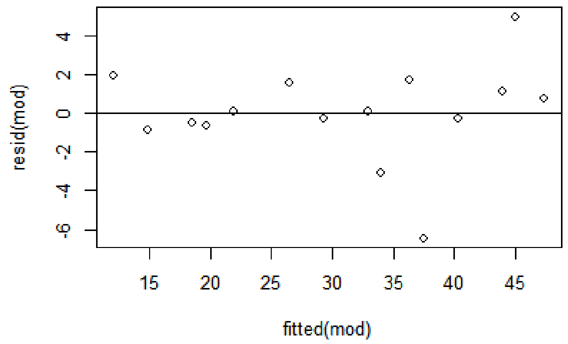


```
> shapiro.test(resid(mod))                ##   p-value = 0.09953 >0.05 GOOD F
OR NORMALITY

        Shapiro-Wilk normality test

data:  resid(mod)
W = 0.90127, p-value = 0.09953

>
> mod <- glmer(npf2 ~ week + (1|cage2), family=poisson, data=data)
> summary(mod)
Generalized linear mixed model fit by maximum likelihood (Laplace Approxima
tion) ['glmerMod']
 Family: poisson  ( log )
Formula: npf2 ~ week + (1 | cage2)
   Data: data

     AIC      BIC   logLik deviance df.resid
    95.3     98.1    -43.7     87.3       11

Scaled residuals:
     Min       1Q   Median       3Q      Max
-1.30053 -0.29629  0.07708  0.27722  0.86257

Random effects:
 Groups Name        Variance Std.Dev.
 cage2  (Intercept) 0.0077   0.08775
Number of obs: 15, groups:  cage2, 5

Fixed effects:
            Estimate Std. Error z value Pr(>|z|)
(Intercept)  3.75270    0.07898  47.515  < 2e-16 ***
```

```
weekWeek 2  -0.29708    0.10465  -2.839  0.00453 **
weekWeek 3  -0.90006    0.12709  -7.082 1.42e-12 ***
---
Signif. codes:  0 '***' 0.001 '**' 0.01 '*' 0.05 '.' 0.1 ' ' 1

Correlation of Fixed Effects:
           (Intr) wekWk2
weekWeek 2 -0.565
weekWeek 3 -0.465  0.351
>
> overdisp_fun <- function(model) {
+   rdf <- df.residual(model)
+   rp <- residuals(model,type="pearson")
+   Pearson.chisq <- sum(rp^2)
+   prat <- Pearson.chisq/rdf
+   pval <- pchisq(Pearson.chisq, df=rdf, lower.tail=FALSE)
+   c(chisq=Pearson.chisq, rdf=rdf, ratio=prat ,p.val=pval)
+ }
> overdisp_fun(mod)                      ###p=0.9480402 >0.05 (no overdispe
rsion)
     chisq        rdf      ratio      p.val
 4.6230540 11.0000000  0.4202776  0.9480402
>
>
> mod2 <- update(mod,~.-week)
> anova(mod,mod2)
Data: data
Models:
mod2: npf2 ~ (1 | cage2)
mod: npf2 ~ week + (1 | cage2)
     npar     AIC     BIC  logLik deviance  Chisq Df Pr(>Chisq)
mod2    2 146.916 148.332 -71.458  142.916
mod     4  95.303  98.136 -43.652   87.303 55.612  2  8.394e-13 ***
---
Signif. codes:  0 '***' 0.001 '**' 0.01 '*' 0.05 '.' 0.1 ' ' 1
> library(multcomp)
Le chargement a nécessité le package : mvtnorm
Le chargement a nécessité le package : survival
Le chargement a nécessité le package : TH.data
Le chargement a nécessité le package : MASS

Attachement du package : 'TH.data'

The following object is masked from 'package:MASS':

    geyser

> summary(glht(mod,linfct = mcp(week="Tukey")))

         Simultaneous Tests for General Linear Hypotheses

Multiple Comparisons of Means: Tukey Contrasts


Fit: glmer(formula = npf2 ~ week + (1 | cage2), data = data, family = poiss
on)

Linear Hypotheses:
                     Estimate Std. Error z value Pr(>|z|)
Week 2 - Week 1 == 0  -0.2971     0.1047  -2.839   0.0123 *
Week 3 - Week 1 == 0  -0.9001     0.1271  -7.082   <0.001 ***
```

```
Week 3 - Week 2 == 0  -0.6030     0.1333  -4.524   <0.001 ***
---
Signif. codes:  0 '***' 0.001 '**' 0.01 '*' 0.05 '.' 0.1 ' ' 1
(Adjusted p values reported -- single-step method)

>
>
> #------------ Plots ----------------#
>
> mean(npf2)
[1] 30.66667
> sd(npf2)/sqrt(nrow(data))
[1] 3.057647
>
> par(mfrow=c(1,2))
> boxplot(npf2~week,ylab="Number of eggs/female", xlab="Egg collection")
> (m<-tapply(npf2,week,mean))
Week 1 Week 2 Week 3
  42.8   31.8   17.4
> (se<-tapply(npf2,week,sd)/sqrt(table(week)))
  Week 1   Week 2   Week 3
3.397058 1.772005 1.536229
> bar<-barplot(m,beside=T,las=1,cex.axis=0.8,ylab="Mean number of eggs/fema
le (±se)", xlab="Egg collection",ylim=c(0,50))
> arrows(bar,m+se,bar,m-se,angle=90,code=3,length=0.05)
>
>
>
> ######################################  END
######################################>



>
############################################################################
#################
> ########################### 2.  Aedes albopictus egg hatching rate
#########################
>
############################################################################
#################
>
>
> #------------ Loading the dataset ---------------------#
>
> library(readr)
> data <- read_delim("~/statsmaiga/MRC optimization/Insect ms
DATA/data_resubm/aedes.egg.hatching.csv",
+                                  ";", escape_double = FALSE, trim_ws =
TRUE)
Parsed with column specification:
cols(
  replicate = col_character(),
  cage = col_character(),
  cage2 = col_character(),
  week = col_character(),
  eggorigin = col_character(),
  eggsample = col_character(),
  hatched = col_double(),
  unhatched = col_double()
)
```

```
> View(data)
> str(data)
tibble [90 x 8] (S3: spec_tbl_df/tbl_df/tbl/data.frame)
 $ replicate: chr [1:90] "R1" "R1" "R1" "R1" ...
 $ cage     : chr [1:90] "C1" "C1" "C1" "C1" ...
 $ cage2    : chr [1:90] "C1" "C1" "C1" "C1" ...
 $ week     : chr [1:90] "Week 1" "Week 1" "Week 1" "Week 1" ...
 $ eggorigin: chr [1:90] "Paper" "Paper" "Paper" "Floating" ...
 $ eggsample: chr [1:90] "S1" "S2" "S3" "S1" ...
 $ hatched  : num [1:90] 94 89 92 81 92 91 93 92 88 84 ...
 $ unhatched: num [1:90] 6 11 8 19 8 10 9 8 12 16 ...
 - attr(*, "spec")=
  .. cols(
  ..   replicate = col_character(),
  ..   cage = col_character(),
  ..   cage2 = col_character(),
  ..   week = col_character(),
  ..   eggorigin = col_character(),
  ..   eggsample = col_character(),
  ..   hatched = col_double(),
  ..   unhatched = col_double()
  .. )
> data <- transform(data,
+                   replicate=factor(replicate),
+                   cage=factor(cage),
+                   cage2=factor(cage2),
+                   week=factor(week),
+                   eggorigin=factor(eggorigin),
+                   eggsample=factor(eggsample))
> str(data)
'data.frame':	90 obs. of  8 variables:
 $ replicate: Factor w/ 2 levels "R1","R2": 1 1 1 1 1 1 1 1 1 1 ...
 $ cage     : Factor w/ 3 levels "C1","C2","C3": 1 1 1 1 1 1 2 2 2 2 ...
 $ cage2    : Factor w/ 5 levels "C1","C2","C3",..: 1 1 1 1 1 1 2 2 2 2 ...
 $ week     : Factor w/ 3 levels "Week 1","Week 2",..: 1 1 1 1 1 1 1 1 1 1
...
 $ eggorigin: Factor w/ 2 levels "Floating","Paper": 2 2 2 1 1 1 2 2 2 1
...
 $ eggsample: Factor w/ 3 levels "S1","S2","S3": 1 2 3 1 2 3 1 2 3 1 ...
 $ hatched  : num  94 89 92 81 92 91 93 92 88 84 ...
 $ unhatched: num  6 11 8 19 8 10 9 8 12 16 ...
> summary (data)
 replicate cage    cage2       week       eggorigin  eggsample    hatched
unhatched
 R1:54     C1:36   C1:18   Week 1:30   Floating:45   S1:30     Min.
:35.00   Min.   : 4.00
 R2:36     C2:36   C2:18   Week 2:30   Paper   :45   S2:30     1st
Qu.:65.25   1st Qu.:16.00
           C3:18   C3:18   Week 3:30                 S3:30     Median
:74.50   Median :25.50
                   C4:18                                       Mean
:72.74   Mean   :27.46
                   C5:18                                       3rd
Qu.:84.75   3rd Qu.:34.00
                                                               Max.
:96.00   Max.   :65.00
> attach(data)
>
>
> #----------- Statistical analysis --------------------#
>
```

```
>
> y <- cbind(hatched,unhatched)
>
> library(lme4)
> mod <- glmer(y ~  week*eggorigin + (1|cage2), family = "binomial")
> summary(mod)
Generalized linear mixed model fit by maximum likelihood (Laplace
Approximation) ['glmerMod']
 Family: binomial  ( logit )
Formula: y ~ week * eggorigin + (1 | cage2)

     AIC      BIC   logLik deviance df.resid
   823.4    840.9   -404.7    809.4       83

Scaled residuals:
    Min      1Q  Median      3Q     Max
-9.7001 -1.0019  0.0474  1.2987  3.3957

Random effects:
 Groups Name        Variance Std.Dev.
 cage2  (Intercept) 0.3158   0.5619
Number of obs: 90, groups:  cage2, 5

Fixed effects:
                          Estimate Std. Error z value Pr(>|z|)
(Intercept)                1.27618    0.25909   4.926 8.41e-07 ***
weekWeek 2                -0.61002    0.08419  -7.245 4.31e-13 ***
weekWeek 3                -0.45360    0.08501  -5.336 9.51e-08 ***
eggoriginPaper             0.46034    0.09489   4.851 1.23e-06 ***
weekWeek 2:eggoriginPaper -0.39714    0.12397  -3.204  0.00136 **
weekWeek 3:eggoriginPaper -0.07864    0.12698  -0.619  0.53573
---
Signif. codes:  0 '***' 0.001 '**' 0.01 '*' 0.05 '.' 0.1 ' ' 1

Correlation of Fixed Effects:
            (Intr) wekWk2 wekWk3 eggrgP wkW2:P
weekWeek 2  -0.181
weekWeek 3  -0.179  0.549
eggorignPpr -0.159  0.490  0.485
wkW2:ggrgnP  0.122 -0.677 -0.371 -0.765
wkW3:ggrgnP  0.119 -0.367 -0.669 -0.747  0.572
>
> overdisp_fun <- function(model) {
+   rdf <- df.residual(model)
+   rp <- residuals(model,type="pearson")
+   Pearson.chisq <- sum(rp^2)
+   prat <- Pearson.chisq/rdf
+   pval <- pchisq(Pearson.chisq, df=rdf, lower.tail=FALSE)
+   c(chisq=Pearson.chisq, rdf=rdf, ratio=prat ,p.val=pval)
+ }
> overdisp_fun(mod)
       chisq          rdf        ratio        p.val
3.836899e+02 8.300000e+01 4.622770e+00 3.371308e-40
>
> data$obs<- 1:nrow(data)
> str(data)
'data.frame':     90 obs. of  9 variables:
 $ replicate: Factor w/ 2 levels "R1","R2": 1 1 1 1 1 1 1 1 1 1 ...
 $ cage     : Factor w/ 3 levels "C1","C2","C3": 1 1 1 1 1 1 2 2 2 2 ...
 $ cage2    : Factor w/ 5 levels "C1","C2","C3",..: 1 1 1 1 1 1 2 2 2 2 ...
```

```
 $ week     : Factor w/ 3 levels "Week 1","Week 2",..: 1 1 1 1 1 1 1 1 1 1
...
 $ eggorigin: Factor w/ 2 levels "Floating","Paper": 2 2 2 1 1 1 2 2 2 1
...
 $ eggsample: Factor w/ 3 levels "S1","S2","S3": 1 2 3 1 2 3 1 2 3 1 ...
 $ hatched  : num  94 89 92 81 92 91 93 92 88 84 ...
 $ unhatched: num  6 11 8 19 8 10 9 8 12 16 ...
 $ obs      : int  1 2 3 4 5 6 7 8 9 10 ...
> mod <- glmer(y ~  week*eggorigin + (1|cage2) + (1|obs), data=data, family
= "binomial")
> summary(mod)
Generalized linear mixed model fit by maximum likelihood (Laplace
Approximation) ['glmerMod']
 Family: binomial  ( logit )
Formula: y ~ week * eggorigin + (1 | cage2) + (1 | obs)
   Data: data

     AIC      BIC   logLik deviance df.resid
   664.4    684.4   -324.2    648.4       82

Scaled residuals:
     Min       1Q   Median       3Q      Max
-1.65296 -0.27143 -0.01174  0.36409  1.19061

Random effects:
 Groups Name        Variance Std.Dev.
 obs    (Intercept) 0.1827   0.4274
 cage2  (Intercept) 0.3363   0.5799
Number of obs: 90, groups:  obs, 90; cage2, 5

Fixed effects:
                          Estimate Std. Error z value Pr(>|z|)
(Intercept)                 1.3471     0.2899   4.647 3.37e-06 ***
weekWeek 2                 -0.6574     0.1797  -3.658 0.000254 ***
weekWeek 3                 -0.4698     0.1807  -2.601 0.009303 **
eggoriginPaper              0.4867     0.1874   2.598 0.009390 **
weekWeek 2:eggoriginPaper  -0.4133     0.2574  -1.605 0.108431
weekWeek 3:eggoriginPaper  -0.1779     0.2590  -0.687 0.492164
---
Signif. codes:  0 '***' 0.001 '**' 0.01 '*' 0.05 '.' 0.1 ' ' 1

Correlation of Fixed Effects:
            (Intr) wekWk2 wekWk3 eggrgP wkW2:P
weekWeek 2  -0.322
weekWeek 3  -0.320  0.516
eggorignPpr -0.308  0.496  0.494
wkW2:ggrgnP  0.224 -0.697 -0.360 -0.728
wkW3:ggrgnP  0.222 -0.359 -0.697 -0.724  0.526
>
> mod2 <- update(mod,~.-week:eggorigin)
> anova(mod,mod2)
Data: data
Models:
mod2: y ~ week + eggorigin + (1 | cage2) + (1 | obs)
mod: y ~ week * eggorigin + (1 | cage2) + (1 | obs)
     npar    AIC    BIC  logLik deviance  Chisq Df Pr(>Chisq)
mod2    6 662.93 677.93 -325.47   650.93
mod     8 664.36 684.36 -324.18   648.36 2.5688  2     0.2768
```

(P >0.05, meaning that the week*eggorigin interaction does not explain the variability in egg hatch) and so the model without the interaction (mod2) is better than mod based also on the AIC)

```
> summary(mod2)
Generalized linear mixed model fit by maximum likelihood (Laplace
Approximation) ['glmerMod']
 Family: binomial  ( logit )
Formula: y ~ week + eggorigin + (1 | cage2) + (1 | obs)
   Data: data

     AIC      BIC   logLik deviance df.resid
   662.9    677.9   -325.5    650.9       84

Scaled residuals:
     Min       1Q   Median       3Q      Max
-1.69115 -0.25583  0.00771  0.31760  1.12212

Random effects:
 Groups Name        Variance Std.Dev.
 obs    (Intercept) 0.1900   0.4359
 cage2  (Intercept) 0.3358   0.5795
Number of obs: 90, groups:  obs, 90; cage2, 5

Fixed effects:
               Estimate Std. Error z value Pr(>|z|)
(Intercept)      1.4463     0.2807   5.152 2.58e-07 ***
weekWeek 2      -0.8601     0.1307  -6.582 4.65e-11 ***
weekWeek 3      -0.5552     0.1314  -4.225 2.39e-05 ***
eggoriginPaper   0.2819     0.1059   2.662  0.00777 **
---
Signif. codes:  0 '***' 0.001 '**' 0.01 '*' 0.05 '.' 0.1 ' ' 1

Correlation of Fixed Effects:
            (Intr) wekWk2 wekWk3
weekWeek 2  -0.244
weekWeek 3  -0.243  0.526
eggorignPpr -0.181 -0.014 -0.014
>
> mod3 <- update(mod2,~.-eggorigin)
> anova(mod2,mod3)
Data: data
Models:
mod3: y ~ week + (1 | cage2) + (1 | obs)
mod2: y ~ week + eggorigin + (1 | cage2) + (1 | obs)
     npar    AIC    BIC  logLik deviance  Chisq Df Pr(>Chisq)
mod3    5 667.75 680.25 -328.87   657.75
mod2    6 662.93 677.93 -325.47   650.93 6.8162  1   0.009033 **
---
Signif. codes:  0 '***' 0.001 '**' 0.01 '*' 0.05 '.' 0.1 ' ' 1
>
> mod4 <- update(mod2,~.-week)
> anova(mod2,mod4)
Data: data
Models:
mod4: y ~ eggorigin + (1 | cage2) + (1 | obs)
mod2: y ~ week + eggorigin + (1 | cage2) + (1 | obs)
     npar    AIC    BIC  logLik deviance  Chisq Df Pr(>Chisq)
mod4    4 695.46 705.46 -343.73   687.46
mod2    6 662.93 677.93 -325.47   650.93 36.528  2   1.17e-08 ***
---
```

```
Signif. codes:  0 ‘***’ 0.001 ‘**’ 0.01 ‘*’ 0.05 ‘.’ 0.1 ‘ ’ 1
>
> library(multcomp)
> summary(glht(mod2,linfct = mcp(week="Tukey")))

       Simultaneous Tests for General Linear Hypotheses

Multiple Comparisons of Means: Tukey Contrasts


Fit: glmer(formula = y ~ week + eggorigin + (1 | cage2) + (1 | obs),
    data = data, family = "binomial")

Linear Hypotheses:
                     Estimate Std. Error z value Pr(>|z|)
Week 2 - Week 1 == 0  -0.8601     0.1307  -6.582   <0.001 ***
Week 3 - Week 1 == 0  -0.5552     0.1314  -4.225   <0.001 ***
Week 3 - Week 2 == 0   0.3049     0.1276   2.389   0.0445 *
---
Signif. codes:  0 ‘***’ 0.001 ‘**’ 0.01 ‘*’ 0.05 ‘.’ 0.1 ‘ ’ 1
(Adjusted p values reported -- single-step method)

> library(emmeans)
> summary(emmeans(mod2,pairwise~week), infer=TRUE, type="response")
$emmeans
 week    prob     SE  df asymp.LCL asymp.UCL z.ratio p.value
 Week 1 0.830 0.0389 Inf     0.740     0.894 5.749   <.0001
 Week 2 0.674 0.0602 Inf     0.547     0.780 2.652   0.0080
 Week 3 0.737 0.0532 Inf     0.621     0.828 3.758   0.0002

Results are averaged over the levels of: eggorigin
Confidence level used: 0.95
Intervals are back-transformed from the logit scale
Tests are performed on the logit scale

$contrasts
 contrast        odds.ratio     SE  df asymp.LCL asymp.UCL z.ratio p.value
 Week 1 / Week 2      2.363 0.3089 Inf     1.740     3.210  6.582  <.0001
 Week 1 / Week 3      1.742 0.2289 Inf     1.280     2.371  4.225  0.0001
 Week 2 / Week 3      0.737 0.0941 Inf     0.547     0.994 -2.389  0.0445

Results are averaged over the levels of: eggorigin
Confidence level used: 0.95
Conf-level adjustment: tukey method for comparing a family of 3 estimates
Intervals are back-transformed from the log odds ratio scale
P value adjustment: tukey method for comparing a family of 3 estimates
Tests are performed on the log odds ratio scale

> summary(emmeans(mod2,pairwise~eggorigin), infer=TRUE, type="response")
$emmeans
 eggorigin  prob     SE  df asymp.LCL asymp.UCL z.ratio p.value
 Floating  0.726 0.0536 Inf     0.610     0.818 3.615   0.0003
 Paper     0.778 0.0466 Inf     0.674     0.856 4.654   <.0001

Results are averaged over the levels of: week
Confidence level used: 0.95
Intervals are back-transformed from the logit scale
Tests are performed on the logit scale

$contrasts
 contrast         odds.ratio     SE  df asymp.LCL asymp.UCL z.ratio p.value
```

```
 Floating / Paper      0.754 0.0799 Inf     0.613     0.928 -2.662  0.0078

Results are averaged over the levels of: week
Confidence level used: 0.95
Intervals are back-transformed from the log odds ratio scale
Tests are performed on the log odds ratio scale

>
>
> #------------ Plots ----------------#
>
> (prop<-sum(hatched)/sum(hatched+unhatched))
[1] 0.7259925
> (IC<-1.96*sqrt(prop*(1-prop)/nrow(data)))
[1] 0.0921472
>
> par(mfrow=c(1,2))
> (p<-tapply(hatched,week,sum)/tapply(hatched+unhatched,week,sum))
   Week 1    Week 2    Week 3
0.8011948 0.6575616 0.7189477
> (IC<-1.96*sqrt(p*(1-p)/tapply(hatched+unhatched,week,sum)))
    Week 1     Week 2     Week 3
0.01425081 0.01697502 0.01607758
> bar<-barplot(p,beside=T,las=1,cex.axis=0.8,ylab="Egg hatch rate
(±95%CI)",
+              xlab="Egg collection", ylim=c(0,1), cex.names=0.9,cex.lab=1)
> arrows(bar,p+IC,bar,p-IC,angle=90,code=3,length=0.05)
>
> (p<-
tapply(hatched,eggorigin,sum)/tapply(hatched+unhatched,eggorigin,sum))
 Floating     Paper
0.7001330 0.7518864
> (IC<-1.96*sqrt(p*(1-p)/tapply(hatched+unhatched,eggorigin,sum)))
  Floating      Paper
0.01336984 0.01261136
> bar<-barplot(p,beside=T,las=1,cex.axis=0.8,ylab="Egg hatch rate
(±95%CI)",
+              xlab="Egg origin", ylim=c(0,1), cex.names=0.9,cex.lab=1)
> arrows(bar,p+IC,bar,p-IC,angle=90,code=3,length=0.05)
>
>
> > #Full model and odds ratio calculation
> mod <- glmer(y ~  week*eggorigin + (1|cage2) + (1|obs), data=data, family
= "binomial")
> summary(mod)

Generalized linear mixed model fit by maximum likelihood (Laplace
Approximation) [
glmerMod]
 Family: binomial  ( logit )
Formula: y ~ week * eggorigin + (1 | cage2) + (1 | obs)
   Data: data

     AIC      BIC   logLik deviance df.resid
   664.4    684.4   -324.2    648.4       82

Scaled residuals:
     Min       1Q   Median       3Q      Max
-1.65296 -0.27143 -0.01174  0.36409  1.19061

Random effects:
```

```
 Groups Name        Variance Std.Dev.
 obs    (Intercept) 0.1827   0.4274
 cage2  (Intercept) 0.3363   0.5799
Number of obs: 90, groups:  obs, 90; cage2, 5

Fixed effects:
                          Estimate Std. Error z value Pr(>|z|)
(Intercept)                 1.3471     0.2899   4.647 3.37e-06 ***
weekWeek 2                 -0.6574     0.1797  -3.658 0.000254 ***
weekWeek 3                 -0.4698     0.1807  -2.601 0.009303 **
eggoriginPaper              0.4867     0.1874   2.598 0.009390 **
weekWeek 2:eggoriginPaper  -0.4133     0.2574  -1.605 0.108431
weekWeek 3:eggoriginPaper  -0.1779     0.2590  -0.687 0.492164
---
Signif. codes:  0 '***' 0.001 '**' 0.01 '*' 0.05 '.' 0.1 ' ' 1

Correlation of Fixed Effects:
            (Intr) wekWk2 wekWk3 eggrgP wkW2:P
weekWeek 2  -0.322
weekWeek 3  -0.320  0.516
eggorignPpr -0.308  0.496  0.494
wkW2:ggrgnP  0.224 -0.697 -0.360 -0.728
wkW3:ggrgnP  0.222 -0.359 -0.697 -0.724  0.526
> # OR
> exp(cbind(OR=fixef(mod)))
                                 OR
(Intercept)               3.8464423
weekWeek 2                0.5181827
weekWeek 3                0.6251050
eggoriginPaper            1.6268830
weekWeek 2:eggoriginPaper 0.6614930
weekWeek 3:eggoriginPaper 0.8370039
> #95% CI
> exp(confint(mod,metho="Wald"))
                              2.5 %    97.5 %
.sig01                           NA        NA
.sig02                           NA        NA
(Intercept)               2.1791154 6.7895066
weekWeek 2                0.3643333 0.7369990
weekWeek 3                0.4387107 0.8906924
eggoriginPaper            1.1268761 2.3487484
weekWeek 2:eggoriginPaper 0.3993896 1.0956042
weekWeek 3:eggoriginPaper 0.5037707 1.3906634
>
#############################################################################################
> #######   3.  Aedes albopictus egg floating rate
#############################################################################################
>
>
> #------------dataset ---------------------#
> library(readr)
> data <- read_delim("~/statsmaiga/MRC optimization/Insect ms
DATA/data_resubm/aedes.egg.floating.csv",
+                                   ";", escape_double = FALSE, trim_ws =
TRUE)
Parsed with column specification:
cols(
  replicate = col_character(),
  cage = col_character(),
```

```
  cage2 = col_character(),
  week = col_character(),
  number = col_double(),
  floating = col_double(),
  paper = col_double()
)
> View(data)
> str(data)
tibble [15 x 7] (S3: spec_tbl_df/tbl_df/tbl/data.frame)
 $ replicate: chr [1:15] "R1" "R1" "R1" "R1" ...
 $ cage     : chr [1:15] "C1" "C2" "C3" "C1" ...
 $ cage2    : chr [1:15] "C1" "C2" "C3" "C1" ...
 $ week     : chr [1:15] "Week 1" "Week 1" "Week 1" "Week 2" ...
 $ number   : num [1:15] 662149 606178 643564 413393 439849 ...
 $ floating : num [1:15] 25796 20853 20354 20325 19139 ...
 $ paper    : num [1:15] 636353 585325 623211 393068 420711 ...
 - attr(*, "spec")=
  .. cols(
  ..   replicate = col_character(),
  ..   cage = col_character(),
  ..   cage2 = col_character(),
  ..   week = col_character(),
  ..   number = col_double(),
  ..   floating = col_double(),
  ..   paper = col_double()
  .. )
> data <- transform(data,
+                   replicate=factor(replicate),
+                   cage=factor(cage),
+                   cage2=factor(cage2),
+                   week=factor(week))
> str(data)
'data.frame':     15 obs. of  7 variables:
 $ replicate: Factor w/ 2 levels "R1","R2": 1 1 1 1 1 1 1 1 1 2 ...
 $ cage     : Factor w/ 3 levels "C1","C2","C3": 1 2 3 1 2 3 1 2 3 1 ...
 $ cage2    : Factor w/ 5 levels "C1","C2","C3",..: 1 2 3 1 2 3 1 2 3 4 ...
 $ week     : Factor w/ 3 levels "Week 1","Week 2",..: 1 1 1 2 2 2 3 3 3 1
...
 $ number   : num  662149 606178 643564 413393 439849 ...
 $ floating : num  25796 20853 20354 20325 19139 ...
 $ paper    : num  636353 585325 623211 393068 420711 ...
> summary (data)
 replicate cage   cage2      week       number          floating
paper
 R1:9      C1:6   C1:3   Week 1:5   Min.   :189282   Min.   : 6198   Min.
:183084
 R2:6      C2:6   C2:3   Week 2:5   1st Qu.:275896   1st Qu.:11404   1st
Qu.:262934
           C3:3   C3:3   Week 3:5   Median :413393   Median :19139   Median
:391225
                  C4:3              Mean   :411882   Mean   :16627   Mean
:395255
                  C5:3              3rd Qu.:525442   3rd Qu.:20604   3rd
Qu.:502132
                                    Max.   :662149   Max.   :26482   Max.
:636353
> attach(data)
The following objects are masked from data (pos = 3):

    cage, week
```

```
The following object is masked from data (pos = 6):

    cage

The following objects are masked from data (pos = 7):

    cage, cage2, replicate, week

> y <- cbind(floating,paper)
> library(lme4)
> mod <- glmer(y ~  week + (1|cage2),family=binomial)
> summary(mod)
Generalized linear mixed model fit by maximum likelihood (Laplace
Approximation) ['glmerMod']
 Family: binomial  ( logit )
Formula: y ~ week + (1 | cage2)

     AIC      BIC   logLik deviance df.resid
  8148.6   8151.4  -4070.3   8140.6       11

Scaled residuals:
    Min      1Q  Median      3Q     Max
-30.819 -18.321  -6.241  22.532  37.528

Random effects:
 Groups Name        Variance Std.Dev.
 cage2  (Intercept) 0.00226  0.04754
Number of obs: 15, groups:  cage2, 5

Fixed effects:
             Estimate Std. Error  z value Pr(>|z|)
(Intercept) -3.163109   0.021475 -147.295  < 2e-16 ***
weekWeek 2  -0.027547   0.004628   -5.953 2.64e-09 ***
weekWeek 3   0.033595   0.005499    6.109 1.00e-09 ***
---
Signif. codes:  0 '***' 0.001 '**' 0.01 '*' 0.05 '.' 0.1 ' ' 1

Correlation of Fixed Effects:
           (Intr) wekWk2
weekWeek 2 -0.091
weekWeek 3 -0.076  0.354
> overdisp_fun <- function(model) {
+   rdf <- df.residual(model)
+   rp <- residuals(model,type="pearson")
+   Pearson.chisq <- sum(rp^2)
+   prat <- Pearson.chisq/rdf
+   pval <- pchisq(Pearson.chisq, df=rdf, lower.tail=FALSE)
+   c(chisq=Pearson.chisq, rdf=rdf, ratio=prat ,p.val=pval)
+ }
> overdisp_fun(mod)
    chisq       rdf     ratio     p.val
8027.3343   11.0000  729.7577    0.0000
> data$obs<- 1:nrow(data)
> str(data)
'data.frame':     15 obs. of  8 variables:
 $ replicate: Factor w/ 2 levels "R1","R2": 1 1 1 1 1 1 1 1 1 2 ...
 $ cage     : Factor w/ 3 levels "C1","C2","C3": 1 2 3 1 2 3 1 2 3 1 ...
 $ cage2    : Factor w/ 5 levels "C1","C2","C3",..: 1 2 3 1 2 3 1 2 3 4 ...
 $ week     : Factor w/ 3 levels "Week 1","Week 2",..: 1 1 1 2 2 2 3 3 3 1
...
 $ number   : num  662149 606178 643564 413393 439849 ...
```

```
 $ floating : num  25796 20853 20354 20325 19139 ...
 $ paper    : num  636353 585325 623211 393068 420711 ...
 $ obs      : int  1 2 3 4 5 6 7 8 9 10 ...
> mod <- glmer(y ~  week + (1|cage2) + (1|obs), data=data, family =
"binomial")
boundary (singular) fit: see ?isSingular
> summary(mod)
Generalized linear mixed model fit by maximum likelihood (Laplace
Approximation) ['glmerMod']
 Family: binomial  ( logit )
Formula: y ~ week + (1 | cage2) + (1 | obs)
   Data: data

     AIC      BIC   logLik deviance df.resid
   289.7    293.2   -139.8    279.7       10

Scaled residuals:
      Min        1Q    Median        3Q       Max
-0.082513 -0.044417 -0.006606  0.031007  0.063308

Random effects:
 Groups Name        Variance Std.Dev.
 obs    (Intercept) 0.03412  0.1847
 cage2  (Intercept) 0.00000  0.0000
Number of obs: 15, groups:  obs, 15; cage2, 5

Fixed effects:
            Estimate Std. Error z value Pr(>|z|)
(Intercept) -3.15604    0.08267 -38.178   <2e-16 ***
weekWeek 2  -0.05903    0.11693  -0.505    0.614
weekWeek 3  -0.01050    0.11697  -0.090    0.928
---
Signif. codes:  0 '***' 0.001 '**' 0.01 '*' 0.05 '.' 0.1 ' ' 1

Correlation of Fixed Effects:
           (Intr) wekWk2
weekWeek 2 -0.707
weekWeek 3 -0.707  0.500
convergence code: 0
boundary (singular) fit: see ?isSingular

> mod2 <- update(mod,~.-week)
boundary (singular) fit: see ?isSingular
> anova(mod,mod2)
Data: data
Models:
mod2: y ~ (1 | cage2) + (1 | obs)
mod: y ~ week + (1 | cage2) + (1 | obs)
     npar    AIC    BIC  logLik deviance  Chisq Df Pr(>Chisq)
mod2    3 285.94 288.07 -139.97   279.94
mod     5 289.66 293.20 -139.83   279.66 0.2872  2     0.8662
> (prop<-sum(floating)/sum(floating+paper))
[1] 0.04036944
> (IC<-1.96*sqrt(prop*(1-prop)/sum(floating+paper)))
[1] 0.0001552037
> (p<-tapply(floating,week,sum)/tapply(floating+paper,week,sum))
    Week 1     Week 2     Week 3
0.04049197 0.03941335 0.04180587
> (IC<-1.96*sqrt(p*(1-p)/tapply(floating+paper,week,sum)))
      Week 1       Week 2       Week 3
0.0002281891 0.0002609939 0.0003616407
```

```
> bar<-barplot(p,beside=T,las=1,cex.axis=0.8,ylab="Egg floating rate
(±95%CI)",
+               xlab="Egg collection", ylim=c(0,1), cex.names=0.9,cex.lab=1)
> arrows(bar,p+IC,bar,p-IC,angle=90,code=3,length=0.05)
>
> #####################################  END
###################################
>
##########################################################################
#################
> ### 4.  Aedes adult mortality rate   #################################
>
##########################################################################
#################
>
>
> #------------ Loading the dataset ---------------------#
> library(readr)
> data <- read_delim("~/statsmaiga/MRC optimization/Insect ms
DATA/data_resubm/aedes.daily.mortality.csv",
+                                    ";", escape_double = FALSE, trim_ws =
TRUE)
Parsed with column specification:
cols(
  cage = col_character(),
  square = col_double(),
  sex = col_character(),
  dailymortality = col_double()
)
> View(data)
> str(data)
tibble [36 x 4] (S3: spec_tbl_df/tbl_df/tbl/data.frame)
 $ cage          : chr [1:36] "C1" "C1" "C1" "C1" ...
 $ square        : num [1:36] 1 2 3 4 5 6 1 2 3 4 ...
 $ sex           : chr [1:36] "Male" "Male" "Male" "Male" ...
 $ dailymortality: num [1:36] 0.058 0.0697 0.1524 0.1131 0.1039 ...
 - attr(*, "spec")=
  .. cols(
  ..   cage = col_character(),
  ..   square = col_double(),
  ..   sex = col_character(),
  ..   dailymortality = col_double()
  .. )
> data$cage <- as.factor(data$cage)
> data$square <- as.factor(data$square)
> data$sex <- as.factor(data$sex)
> str(data)
tibble [36 x 4] (S3: spec_tbl_df/tbl_df/tbl/data.frame)
 $ cage          : Factor w/ 3 levels "C1","C2","C3": 1 1 1 1 1 1 1 1 1 1
...
 $ square        : Factor w/ 6 levels "1","2","3","4",..: 1 2 3 4 5 6 1 2 3
4 ...
 $ sex           : Factor w/ 2 levels "Female","Male": 2 2 2 2 2 2 1 1 1 1
...
 $ dailymortality: num [1:36] 0.058 0.0697 0.1524 0.1131 0.1039 ...
 - attr(*, "spec")=
  .. cols(
  ..   cage = col_character(),
  ..   square = col_double(),
  ..   sex = col_character(),
  ..   dailymortality = col_double()
```

```
  .. )
> summary (data)
 cage    square     sex     dailymortality
 C1:12   1:6    Female:18   Min.   :0.05795
 C2:12   2:6    Male  :18   1st Qu.:0.10358
 C3:12   3:6                Median :0.11343
         4:6                Mean   :0.11456
         5:6                3rd Qu.:0.12875
         6:6                Max.   :0.15718
> attach(data)
The following object is masked from data (pos = 3):

    cage

> library(lme4)
> mod <- lmer(dailymortality ~ sex + (1|cage))
> summary(mod)
Linear mixed model fit by REML ['lmerMod']
Formula: dailymortality ~ sex + (1 | cage)

REML criterion at convergence: -154.6

Scaled residuals:
     Min       1Q   Median       3Q      Max
-2.39150 -0.44717 -0.00462  0.67424  1.85751

Random effects:
 Groups   Name        Variance  Std.Dev.
 cage     (Intercept) 9.113e-06 0.003019
 Residual             5.180e-04 0.022760
Number of obs: 36, groups:  cage, 3

Fixed effects:
             Estimate Std. Error t value
(Intercept)  0.115449   0.005641  20.468
sexMale     -0.001781   0.007587  -0.235

Correlation of Fixed Effects:
        (Intr)
sexMale -0.673
> par(mfrow=c(2,2))
> hist(resid(mod),freq=FALSE, ylim = c())
> lines(density(resid(mod)), col="red")
> library(car)
Le chargement a nécessité le package : carData
Registered S3 methods overwritten by 'car':
  method                          from
  influence.merMod                lme4
  cooks.distance.influence.merMod lme4
  dfbeta.influence.merMod         lme4
  dfbetas.influence.merMod        lme4
> qqPlot(resid(mod))  # good for normality
[1] 1 2
> plot(fitted(mod),resid(mod))
> abline(h=0)  # good for variance
```

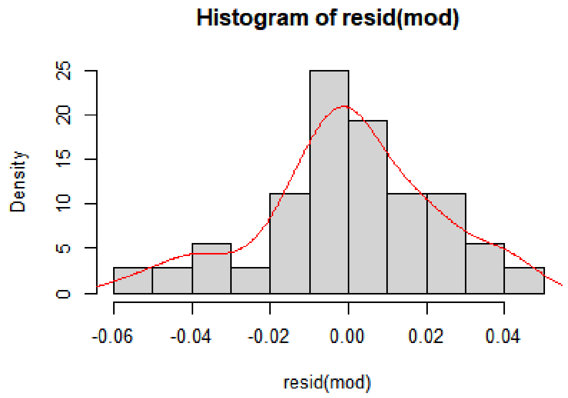

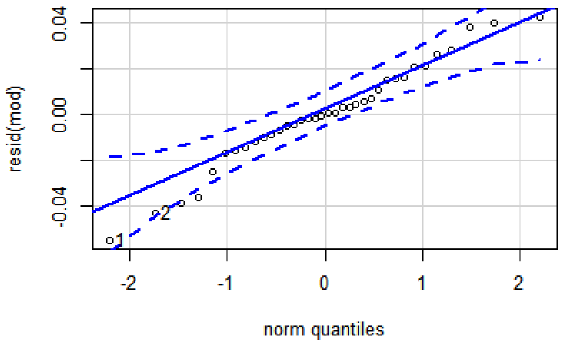

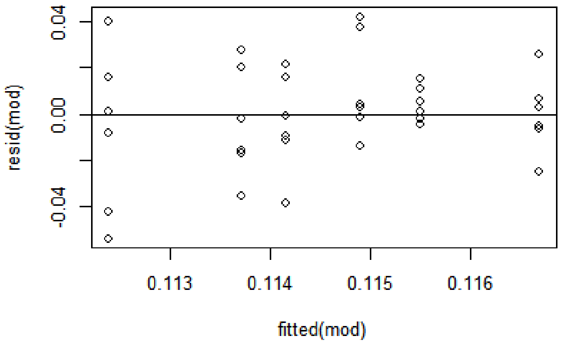


```
> shapiro.test(resid(mod))     # p-value = 0.5383  good for normality

	Shapiro-Wilk normality test

data:  resid(mod)
W = 0.97381, p-value = 0.5383

> mod2 <- update(mod,~.-sex)
> anova(mod,mod2)
refitting model(s) with ML (instead of REML)
Data: NULL
Models:
mod2: dailymortality ~ (1 | cage)
mod: dailymortality ~ sex + (1 | cage)
     npar     AIC     BIC logLik deviance  Chisq Df Pr(>Chisq)
mod2    3 -165.75 -161.00 85.876  -171.75
mod     4 -163.81 -157.47 85.904  -171.81 0.0576  1     0.8103>>
```

```
################################################################################
#################
########################### 5.  Anopheles arabiensis egg production
#########################
################################################################################
#################


#------------ Loading the dataset ---------------------#

library(readr)
data <- read_delim("~/statsmaiga/MRC optimization/Insect ms
DATA/data_resubm/anopheles.egg.production.csv",
                                        ";", escape_double = FALSE, trim_ws
= TRUE)
View(data)

str(data)
data <- transform(data,
                  cage=factor(cage),
                  week=factor(week),
                  eggbatch=factor(eggbatch),
                  cagetype=factor(cagetype))
str(data)
summary (data)
attach(data)

library(lme4)
mod <- lmer(npf2 ~ cagetype*eggbatch + (1|cage))
summary(mod)

par(mfrow=c(2,2))
hist(resid(mod),freq=FALSE, ylim = c(0,0.3))
lines(density(resid(mod)), col="red")
library(car)
qqPlot(resid(mod))
plot(fitted(mod),resid(mod))
abline(h=0)
```

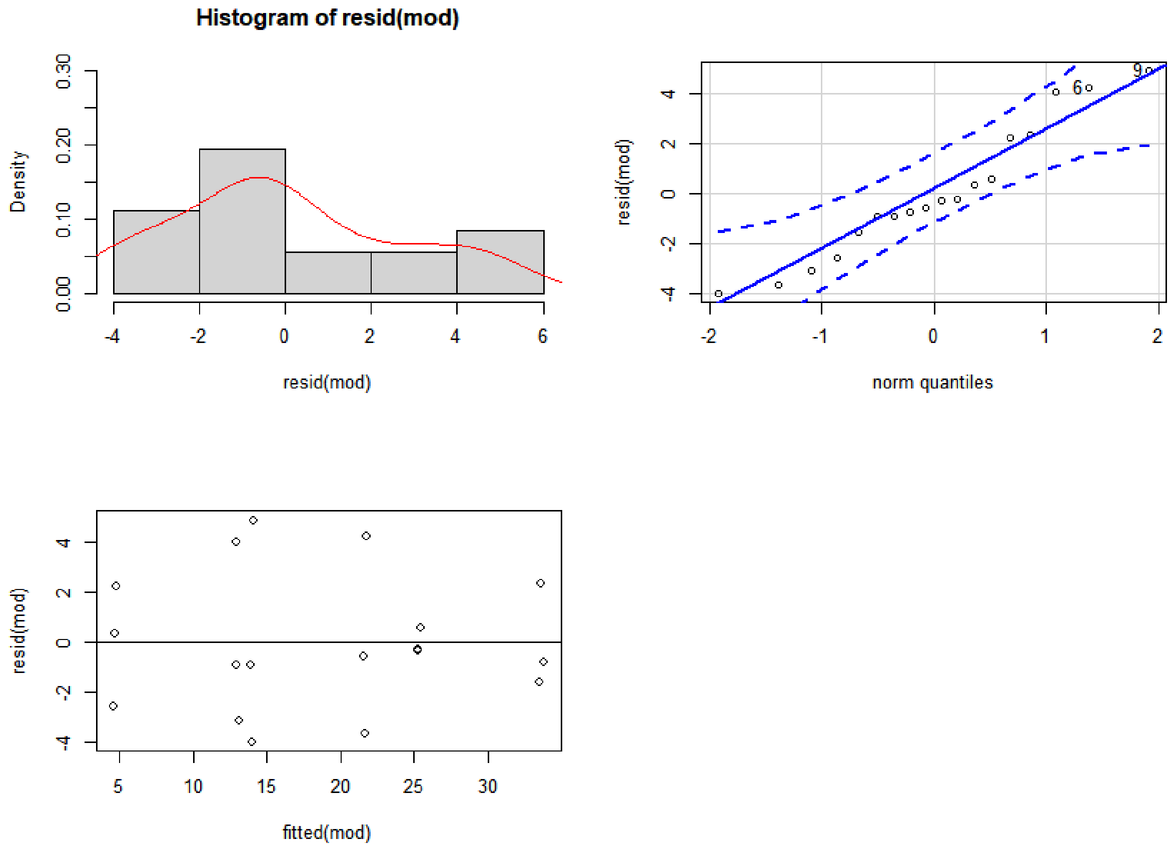


```
shapiro.test(resid(mod))     # p-value = 0.3534   # good

mod <- glmer(npf2 ~ cagetype*eggbatch + (1|cage), family=poisson)
summary(mod)

overdisp_fun <- function(model) {
  rdf <- df.residual(model)
  rp <- residuals(model,type="pearson")
  Pearson.chisq <- sum(rp^2)
  prat <- Pearson.chisq/rdf
  pval <- pchisq(Pearson.chisq, df=rdf, lower.tail=FALSE)
  c(chisq=Pearson.chisq, rdf=rdf, ratio=prat ,p.val=pval)
}
overdisp_fun(mod)

#------------ Statistical analysis ---------------------#


mod2 <- update(mod,~.-cagetype:eggbatch)
anova(mod,mod2)
library(car)
Anova(mod,Type=III)

library(multcomp)
summary(glht(mod,linfct = mcp(eggbatch="Tukey")))
library(emmeans)
summary(emmeans(mod,pairwise~cagetype|eggbatch),infer=TRUE,
type="response")

#------------ Plots ---------------#
```

```
mean(neggs)
sd(neggs)/sqrt(nrow(data))

par(mfrow=c(1,2))
boxplot(npf2 ~ cagetype,ylab="Number of eggs/female", xlab="Cage type")

(m<-tapply(npf2,cagetype,mean))
(se<-tapply(npf2,cagetype,sd)/sqrt(table(cagetype)))
bar<-barplot(m,beside=T,las=1,cex.axis=0.8,ylab="Mean number of eggs
/female (±se)", xlab="Cage type",ylim=c(0,35))
arrows(bar,m+se,bar,m-se,angle=90,code=3,length=0.05)

boxplot(npf2 ~ eggbatch,ylab="Number of eggs/female", xlab="Egg batch")
(m<-tapply(npf2,eggbatch,mean))
(se<-tapply(npf2,eggbatch,sd)/sqrt(table(eggbatch)))
bar<-barplot(m,beside=T,las=1,cex.axis=0.8,ylab="Mean number of eggs/female
(±se)", xlab="Egg collection",ylim=c(0,35))
arrows(bar,m+se,bar,m-se,angle=90,code=3,length=0.05)

boxplot(npf2~cagetype:eggbatch, sep = "_", lex.order = TRUE, col =
c("gray25", "gray70","gray90"),ylab="Number of eggs produced by female",
xlab="Egg batch")
(m<-tapply(npf2,list(eggbatch,cagetype),mean))
(se<-
tapply(npf2,list(eggbatch,cagetype),sd)/sqrt(table(eggbatch,cagetype)))
bar<-barplot(m,beside=T,las=1,cex.axis=0.8,ylab="Mean number of eggs/female
(±se)", xlab=" Egg collection x cage type interaction",ylim=c(0,35))
arrows(bar,m+se,bar,m-se,angle=90,code=3,length=0.05)
legend(6.6,38,levels(eggbatch), fill=c("gray25", "gray70","gray90"),xpd =
T)

(m<-tapply(npf2,list(cagetype,eggbatch),mean))
(se<-
tapply(npf2,list(cagetype,eggbatch),sd)/sqrt(table(cagetype,eggbatch)))
bar<-barplot(m,beside=T,las=1,cex.axis=0.8,ylab="Mean number of eggs/female
(±se)", xlab=" Egg collection : cage type interaction",ylim=c(0,35))
arrows(bar,m+se,bar,m-se,angle=90,code=3,length=0.05)
legend(6.6,38,levels(cagetype), fill=c("gray25", "gray70","gray90"),xpd =
T)


######################################  END
###########################################################################
######################
```
