# Supplementary figures and images for: Assessment of a Novel Adult Mass-Rearing Cage for Aedes albopictus (Skuse) and Anopheles arabiensis (Patton)
Source: Insects. 2020 Nov 13;11(11):801. doi: 10.3390/insects11110801 (PMC7697024; doi:10.3390/insects11110801)

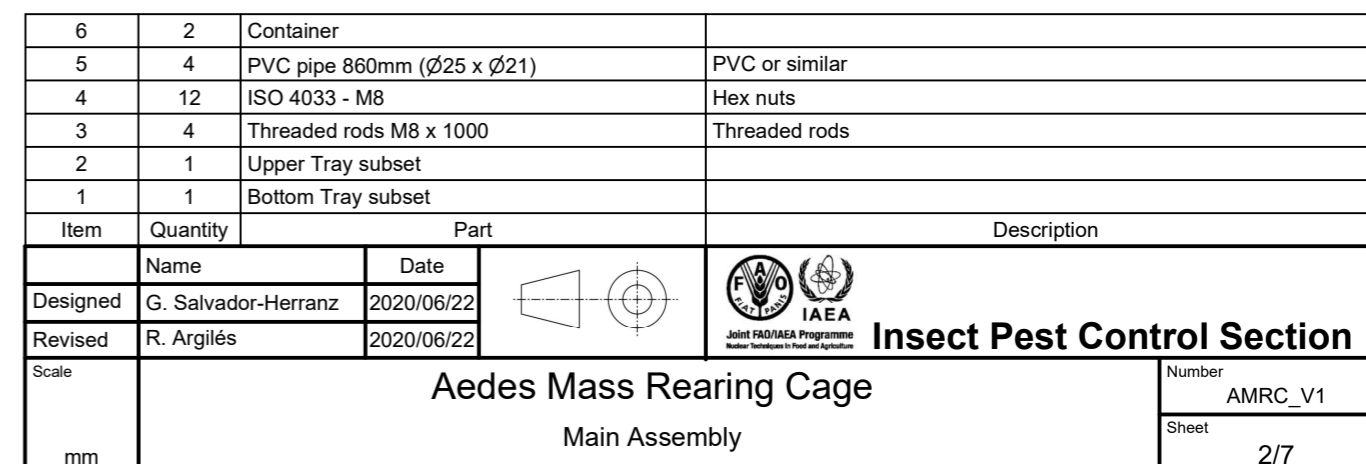

Supplement: Supplementary file 1 [file insects-11-00801-s001.zip › Supplementary Materials/Figure S2. Main_Assembly of the new mass-rearing cage.pdf]
